# Advances in Neuroprotection in Glaucoma: Pharmacological Strategies and Emerging Technologies

**DOI:** 10.3390/ph17101261

**Published:** 2024-09-25

**Authors:** Li-Hsin Wang, Chun-Hao Huang, I-Chan Lin

**Affiliations:** 1 School of Medicine, College of Medicine, Taipei Medical University, Taipei 110301, Taiwan; b101107053@tmu.edu.tw; 2 Department of Ophthalmology, Wan Fang Hospital, Taipei Medical University, Taipei 110301, Taiwan; 110072@w.tmu.edu.tw; 3 Department of Ophthalmology, School of Medicine, College of Medicine, Taipei Medical University, Taipei 11031, Taiwan

**Keywords:** neuroprotection, glaucoma, retinal neurodegeneration

## Abstract

Glaucoma is a major global health concern and the leading cause of irreversible blindness worldwide, characterized by the progressive degeneration of retinal ganglion cells (RGCs) and their axons. This review focuses on the need for neuroprotective strategies in glaucoma management, addressing the limitations of current treatments that primarily target intraocular pressure (IOP) reduction. Despite effective IOP management, many patients continue to experience RGC degeneration, leading to irreversible blindness. This review provides an overview of both pharmacological interventions and emerging technologies aimed at directly protecting RGCs and the optic nerve, independent of IOP reduction. Pharmacological agents such as brimonidine, neurotrophic factors, memantine, Ginkgo biloba extract, citicoline, nicotinamide, insulin, and resveratrol show promise in preclinical and early clinical studies for their neuroprotective properties. Emerging technologies, including stem cell therapy, gene therapy, mitochondrial-targeted therapies, and nanotechnologies, offer innovative approaches for neuroprotection and regeneration of damaged RGCs. While these interventions hold significant potential, further research and clinical trials are necessary to confirm their efficacy and establish their role in clinical practice. This review highlights the multifaceted nature of neuroprotection in glaucoma, aiming to guide future research and clinical practice toward more effective management of glaucoma-induced neurodegeneration.

## 1. Introduction

Glaucoma is a major global health concern and is recognized as one of the primary causes of irreversible blindness worldwide. The condition is typified by the gradual deterioration of retinal ganglion cells (RGCs) and their axons, resulting in the loss of the visual field and ultimately irreversible blindness. With over 70 million individuals affected globally, the impact of glaucoma is substantial and is predicted to increase to 111.8 million affected individuals by 2040 [1].

While current treatments primarily focus on lowering intraocular pressure (IOP), RGC loss can occur even with normal IOP levels, and a significant proportion of patients continue to experience vision loss despite effective IOP management. There is increasing evidence that other mechanisms, such as oxidative stress, excitotoxicity, vascular dysregulation, and neuroinflammation, also play a significant role in the pathogenesis of the disease [2,3,4]. This has led to a growing interest in neuroprotective strategies that aim to directly preserve and protect RGCs and the optic nerve from neurodegenerative processes, independent of reducing IOP, therefore slowing down the decline in visual function. By focusing on neuroprotection, researchers hope to develop therapies that can halt or even reverse the damage to the optic nerve, providing a significant advancement in the management of glaucoma.

This review aims to provide a comprehensive overview of the current state of neuroprotective strategies for glaucoma, focusing on both pharmacological treatments and emerging technologies. By examining a wide range of approaches, this review seeks to highlight the multifaceted nature of neuroprotection. This holistic perspective is essential for understanding the potential benefits and limitations of each strategy, guiding future research and clinical practice towards a more effective management of glaucoma-induced neurodegeneration.

## 2. Mechanisms of Loss of Retinal Functions in Glaucoma

**Figure 1:**
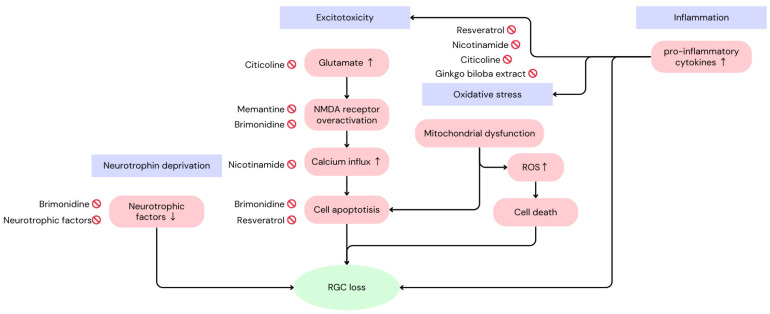
The mechanisms of glaucoma inducing the loss of retinal ganglion cells and the targets of each neuroprotective compound [5].

The key mechanisms underlying RGC death in glaucoma involve a complex interplay of various pathological processes, including neurotrophin deprivation, excitotoxicity, oxidative stress, mitochondrial dysfunction, inflammation, and apoptosis (Figure 1). 

Neurotrophins, such as ciliary neurotrophic factor (CNTF) and brain-derived neurotrophic factor (BDNF), are crucial in the survival and maintenance of RGCs. In glaucoma, the deprivation of these neurotrophins due to impaired axonal transport and receptor expression leads to RGC degeneration [6].

Excitotoxicity, primarily driven by elevated levels of the neurotransmitter glutamate, leads to the overactivation of N-methyl-D-aspartate (NMDA) receptors on RGCs, causing a harmful influx of calcium ions that triggers apoptotic pathways. While the role of glutamate in glaucoma is complicated and not entirely understood, it is clear that its dysregulation contributes significantly to RGC death [2].

Oxidative stress is another critical factor in glaucomatous neurodegeneration. An imbalance between reactive oxygen species (ROS) production and the body’s antioxidant defenses leads to oxidative damage in retinal ganglion cells (RGCs), affecting proteins, lipids, and DNA. This oxidative stress can be exacerbated by mitochondrial dysfunction, as RGCs have high energy demands, and any impairment in mitochondrial function can lead to energy deficits, further compromising cell viability. The release of cytochrome c from damaged mitochondria into the cytoplasm is a key event that activates the apoptotic machinery in RGCs [3].

Inflammation also plays a significant role in glaucoma, with pro-inflammatory cytokines like tumor necrosis factor-alpha (TNF-α) being upregulated in the retina and optic nerve head. These cytokines can induce RGC apoptosis either directly or by exacerbating excitotoxicity and oxidative stress. Additionally, the glial cells in the retina, including astrocytes, Müller cells, and microglia, contribute to both protective and detrimental responses in glaucoma. While they can help clear debris and produce neurotrophic factors, their overactivation can lead to chronic inflammation and further RGC damage [4].

Apoptosis, or programmed cell death, is the final common pathway through which many of these mechanisms converge to cause RGC loss in glaucoma. Both intrinsic and extrinsic apoptotic pathways are activated in response to the various stressors affecting RGCs. Key molecules in these pathways include the Bcl-2 family of proteins, which control mitochondrial membrane permeability, and caspases, which are the executioners of apoptosis [7].

## 3. Pharmacological Interventions (Summary in Table 1)

### 3.1. Brimonidine

Brimonidine, an alpha-2 adrenergic agonist, widely used to reduce IOP, has demonstrated notable neuroprotective properties in the treatment of glaucoma. While its IOP-lowering effects are well documented, emerging evidence highlights its potential to protect RGCs through various mechanisms independent of IOP reduction.

**Table 1:** Summary of neuroprotective compounds for glaucoma with clinical trials.

Active Compound	Mechanism of Action	Route of Administration	Status in Clinical Studies	Clinical Trials
Brimonidine	Binds alpha-2 adrenergic receptors, regulates apoptotic proteins, upregulates neurotrophic factors, reduces NMDA ^1^ receptor excitotoxicity	Topical	FDA-approved for IOP ^2^ reduction, neuroprotective effects under investigation	Krupin et al., 2011 [8]; De Moraes et al., 2012 [9]
Neurotrophic Factors (BDNF ^3^, CNTF ^4^, NGF ^5^)	Binds to specific receptors (TrkB and TrkA), promotes survival of neurons, enhances axon regeneration, modulates apoptosis	Implants (CTNF), topical (rhNGF ^6^)	Ongoing Phase II trials (CTNF), Phase Ib trial showed safety (rhNGF)	Goldberg et al., 2023 [10]; Beykin et al., 2022 [11]
Memantine	Non-competitive NMDA receptor antagonist, reduces calcium influx, protects neurons from glutamate-induced excitotoxicity	Oral	Failed large-scale clinical trials	Weinreb et al., 2018 [12]
GBE ^7^	Scavenges ROS, reduces oxidative stress, stabilizes mitochondria, antagonizes PAF	Oral	Mixed results in clinical trials	Quaranta et al., 2003 [13]; Lee et al., 2013 [14]; Guo et al., 2014 [15]
Citicoline	Serves as precursor for phospholipids, enhances neurotransmitter synthesis	Oral, topical	Ongoing large-scale clinical trials	NCT05315206 NCT05710198
Nicotinamide	Replenishes NAD levels, supports mitochondrial function, regulates calcium homeostasis, reduces oxidative stress	Oral	Ongoing large-scale long-term trials	NCT05275738 NCT05405868

^1^ N-methyl-D-aspartate, ^2^ intraocular pressure, ^3^ brain-derived neurotrophic factor, ^4^ ciliary neurotrophic factor, ^5^ nerve growth factor, ^6^ recombinant human nerve growth factor, ^7^ ginkgo biloba extract.

Brimonidine has a quinoxaline chemical structure with an imidazoline ring that allows it to bind to alpha-2 adrenergic receptors in the eye. Several preclinical studies have explored the neuroprotective mechanisms of brimonidine. It has been shown to enhance the survival of RGCs and protect RGCs from various types of optic nerve injuries, including NMDA-induced neurotoxicity, ischemia, optic neuritis, and ocular hypertension [16,17,18,19,20,21,22,23]. It achieves these protective effects by regulating anti- or pro-apoptotic proteins, such as Bcl-2/BxL or Bax, and upregulating neurotrophic factors like brain-derived neurotrophic factor (BDNF), fibroblast growth factor (FGF), and their receptors [24,25,26]. Brimonidine also alleviates neuronal death by altering NMDA receptors and reducing glutamate accumulation post-injury, thereby decreasing excitotoxicity [27,28,29]. Additionally, brimonidine interferes with the amyloid-β pathway, reducing amyloid-β levels, which are implicated in RGC death [30,31]. Improvement in impaired vascular autoregulation, protection of the retina from ischemic injury, and support of neuronal regeneration were also observed [16,19,20,22,23,24,32,33,34]. These effects suggest that brimonidine’s neuroprotection is not solely reliant on its ability to lower IOP. Recent studies have also shown additive protective effects when combining brimonidine and ripasudil. This combined treatment modulates multiple signaling pathways, including the suppression of proinflammatory mediators and the increase in trophic factors, leading to enhanced RGC survival compared to single-agent treatments [35,36].

Clinical studies have provided mixed but promising results regarding the neuroprotective effects of brimonidine. The Low-pressure Glaucoma Treatment Study (LoGTS) compared the effects of brimonidine and timolol in patients with low-pressure glaucoma. Although both medications were similarly effective in lowering IOP, visual field deterioration was notably less frequent in the brimonidine group compared to the timolol group. However, caution is advised when interpreting the results due to the high dropout rate, especially among those taking brimonidine [8,9]. Topical brimonidine also enhanced contrast sensitivity regardless of its effects on IOP, whereas timolol showed no such improvement [37]. Another study suggests that brimonidine may preserve retinal nerve fiber layer (RNFL) independent of its IOP-lowering effects [38].

Despite these findings, the evidence for brimonidine’s neuroprotective effects in clinical settings remains inconclusive. The high attrition rates and adverse effects reported in some studies raise concerns about the consistency and applicability of these results. Large double-blinded, randomized controlled trials are needed to definitively determine the role of brimonidine in the treatment of glaucoma [39].

### 3.2. Neurotrophic Factors

Neurotrophic factors (NTFs) are essential molecules involved in neuron development, survival, and repair. They bind to specific receptors, activating tyrosine kinase signaling pathways, which lead to various neuroprotective actions, such as the promotion of axon regeneration and enhancement of neuronal cell function [40,41,42].

Neurotrophic factors such as nerve growth factor (NGF), BDNF, CNTF, FGF-2, glial cell-line-derived neurotrophic factor (GDNF), neurturin (NRTN), and neuritin are particularly relevant to glaucoma. These factors have been shown to prevent RGC death in several rodent models of glaucoma [43,44,45,46,47,48,49,50].

BDNF plays a crucial role in the survival of retinal ganglion cells (RGCs) by preventing apoptosis through the activation of extracellular signal-regulated kinases (Erk1/2) and c-jun, while inhibiting caspase 2 via its receptor, TrkB [51,52]. Research has shown significantly reduced levels of BDNF in the serum and aqueous humor of patients with normal tension glaucoma (NTG) and primary open-angle glaucoma (POAG) [44,53,54]. BDNF has also been shown to improve RGC survival and prevent RGC death induced by amyloid-β-triggered apoptosis in rat models [40,55,56]. Nonetheless, further studies are needed to clarify the causal link between BDNF and glaucoma and to assess the effectiveness of BDNF supplementation as a neuroprotective treatment.

CNTF is produced locally by RGCs, and its levels are also found to be decreased in the aqueous humor and lacrimal fluid of patients with POAG [57]. CNTF exerts its effects through a heterotrimeric receptor complex that includes CNTF receptor α, gp130, and leukemia inhibitory factor receptor β [58]. It can protect damaged RGCs directly by binding to receptors on their surface, or indirectly by influencing glial cell activity via the JAK/STAT, PI3K/AKT, and MAPK signaling pathways [59,60,61,62]. Preclinical studies have shown a promising neuroprotective ability of CNTF with single intravitreal injection [63]. The combination of CNTF with other drugs such as cyclic adenosine monophosphate (cAMP) and Rho kinase inhibitor was also investigated, with benefits shown in axon regeneration [64,65,66]. The NT-501 device, a polymer capsule housing a genetically modified human cell line that releases CNTF, has been designed for long-term delivery. Ongoing clinical trials are evaluating the therapeutic effectiveness of NT-501-encapsulated cell therapy in the treatment of glaucoma. Phase I trials showed that NT-501 implants were safe, and ongoing Phase II trials aim to evaluate the efficacy of improving visual fields and retinal structure [10,67,68,69].

NGF promotes the survival and regeneration of retinal and brain cells [70]. It works by interacting with TrkA and p75 neurotrophin receptors (p75NTR). When NGF binds to TrkA, it activates the PI3K/Akt pathway, which boosts Bcl-2 levels and lowers Bax proteins, ultimately inhibiting apoptosis [71]. It also activates the RAS and PLC pathways, which are involved in regulating cell survival and differentiation [72]. Topical application of NGF preserved RGCs and their axons in a model of ocular hypertension [43,73]. It has been shown that in advanced glaucoma patients, topical treatment with NGF improved optic nerve functions, including visual acuity, visual field, and contrast sensitivity [43]. In preclinical studies, recombinant human NGF (rhNGF) enhanced neuroprotection, neuroregeneration, and axon growth were associated with reduced p75NTR and increased TrkA phosphorylation [71,74,75,76]. RhNGF has been evaluated in clinical trials, showing safety in healthy volunteers and some neuroprotective effects in glaucoma patients [11,77].

Despite promising preclinical results, translating these findings into clinical practice poses several challenges. The effective and long-lasting delivery of NTFs to the retina is a significant hurdle. Intravitreal injections, though effective, may not be practical for chronic conditions like glaucoma that require long-term treatment. Future research should focus on long-term studies and innovative delivery systems to fully harness the potential of neurotrophic factors in glaucoma therapy.

### 3.3. Memantine

Memantine is a voltage-dependent, non-competitive NMDA receptor antagonist with low affinity that selectively binds to activated glutamatergic receptors [78,79], reducing excessive glutamatergic activity, calcium influx, and the activation of pro-apoptotic cascades without affecting normal neurotransmission [80,81], thereby protecting RGCs from excitotoxic damage. Its rigid tricyclic amine structure (1-amino-3,5-dimethyladamantane) allows it to effectively block the NMDA receptor channel by interacting with critical asparagine residues (N-sites) like N616 in the NR1 and NR2 subunits. This binding inhibits excessive calcium influx while preserving normal receptor activity. Key amino acids involved in memantine’s channel blockade include N616, A645, and Y647 in NR1, and N615 in NR2B.

In animal models, memantine has demonstrated promising neuroprotective effects. Intraperitoneal, subcutaneous, and oral administrations of memantine in monkeys and rats improve RGC survival, prevent optic nerve fiber loss, and maintain retinal and visual pathway function without adverse effects on normal eyes [80,82,83]. However, the role of elevated glutamate in glaucoma remains debated and inconsistent [84,85,86].

Although preclinical studies have shown promising results, large-scale clinical trials have not been able to confirm significant benefits of memantine in the treatment of glaucoma. Two double-masked, placebo-controlled, multicenter Phase III clinical trials (NCT00141882 and NCT00168350) involving 2296 patients with POAG over four years did not show that memantine delayed visual field progression compared to the placebo [12].

To better evaluate memantine’s potential in glaucoma treatment, future studies should consider including patients with early-stage glaucoma and a less heterogeneous population regarding progression risk factors. Longer study durations, higher doses, and alternative methods of medication delivery should be explored. Additionally, using more advanced imaging techniques could provide more objective structural parameters for assessing early glaucoma progression [12,87].

### 3.4. Ginkgo Biloba Extract

*Ginkgo biloba* has been used in traditional Chinese medicine for hundreds of years. Modern research has explored the neuroprotective properties of Ginkgo biloba extract (GBE), particularly in the context of neurodegenerative diseases like glaucoma [88,89].

GBE contains active compounds such as flavonoid glycosides and terpene lactones, including ginkgolides A, B, C, and bilobalide, which contribute to its neuroprotective effects. The flavonoid glycosides, with their polyphenolic structure with two aromatic rings and glycosidic attachments, exhibit antioxidant properties by scavenging ROS and reducing oxidative stress. Terpene lactones, particularly ginkgolide B with its cage-like structure, antagonize the platelet activator factor (PAF), reducing trauma-induced apoptosis and enhancing recovery from ischemic injury. Bilobalide, another key terpene with its trilactone rings, stabilizes mitochondria, further mitigating ROS production. GBE’s ability to reduce lipid peroxidation helps protect cellular membranes and enhance erythrocyte deformability and blood perfusion. Additionally, both flavonoids and terpenoids in GBE contribute to vasodilation, supporting improved blood flow in tissues, and further reinforcing its neuroprotective properties [90].

In preclinical studies, GBE has demonstrated significant neuroprotective effects, highlighting GBE’s potential to enhance RGC survival and protect against glaucomatous damage [91,92,93]. Clinical trials investigating the effects of GBE on glaucoma patients have yielded mixed results. One study noted improvements in preexisting visual field defects in patients with NTG after one month of taking GBE oral capsules [13]. Another 4-year long-term study found that taking GBE significantly slowed the progression of visual field defects in NTG patients without affecting IOP [14]. However, a randomized controlled trial (RCT) showed no effect on visual field performance or contrast sensitivity in NTG patients of the same GBE dosage and duration as the first study [15], highlighting the variability in clinical outcomes. Numerous studies have shown increased blood flow velocity and decreased vascular resistance in the retrobulbar and peripapillary vascular systems [94,95].

Despite promising preclinical findings, the clinical evidence for GBE’s neuroprotective effects in glaucoma remains inconclusive [90]. The discrepancies in clinical trial results underscore the need for further research to identify the specific conditions and patient populations that may benefit from GBE treatment. Future studies should consider enrolling patients with early-stage glaucoma, controlling for hypotensive treatments, and utilizing advanced imaging techniques. In summary, GBE holds promise as a neuroprotective treatment for glaucoma, but larger, well-designed clinical trials are needed to validate its effectiveness and determine its place in clinical practice.

### 3.5. Citicoline

Citicoline, also known as cytidine 5′-diphosphocholine, a naturally occurring compound, has garnered attention for its potential neuroprotective effects in the context of glaucoma.

The chemical structure of citicoline consists of cytidine linked to a diphosphate bridge and choline, allowing it to serve as a precursor for the synthesis of important phospholipids such as phosphatidylcholine, phosphatidylethanolamine, sphingomyelin, and cardiolipin, which are essential components of neuronal cell membranes [96]. These phospholipids are integral for the formation of synaptic membranes, enhancing the release and recycling of neurotransmitters like acetylcholine, dopamine, serotonin, and norepinephrine, which are crucial for normal neuronal function [97]. The choline component also facilitates the synthesis of acetylcholine. While stabilizing neuronal membranes and enhancing important neurotransmitter synthesis, citicoline reduces glutamate excitotoxicity, improves axonal transport deficits, and lowers oxidative stress by producing glutathione [98].

In animal models, citicoline has demonstrated significant neuroprotective effects. Studies have shown that the administration of citicoline can attenuate the decrease in RGC density and improve visual acuity and the integrity of the pre-chiasmatic white matter of the optic nerve without impacting IOP, indicating a neuroprotective effect [99].

Early studies demonstrated that intramuscular (IM) citicoline improved visual field performance in patients with POAG, with long-term benefits in visual field preservation [100]. Other studies have also shown improvements in visual evoked potentials (VEP) and pattern electroretinograms (PERG) with IM citicoline [101]. Studies evaluating the effects of oral citicoline reported similar benefits on retinocortical function [102,103]. Longitudinal studies have also shown that oral citicoline can slow the progression of glaucomatous damage, with significant improvements in RNFL and ganglion cell complex thickness [104]. The topical administration of citicoline eyedrops has also shown similar effects on retinocortical function, as measured by PERG and VEP [105,106,107,108], but with more adverse effects compared to oral formulation [109]. Other studies investigating the combination therapy of citicoline with drugs including docosahexaenoic acid, homotaurine, and vitamin E also showed significant visual improvement [110,111]. Citicoline-loaded liposomes were also investigated, showing promising results for improved bioavailability and sustained drug release in ocular applications [112].

The neuroprotective potential of citicoline in glaucoma has sparked several ongoing clinical trials to explore its therapeutic benefits. One such trial is a randomized controlled trial evaluating the effects of oral citicoline over a year in patients with OAG (NCT05315206). Furthermore, a large Phase III randomized controlled trial is being planned to determine the efficacy of citicoline eyedrops in slowing visual field deterioration and structural changes in glaucoma patients (NCT05710198).

Citicoline has become a potential neuroprotective treatment for glaucoma. Although initial preclinical and early clinical studies have shown promising outcomes, larger and more thorough trials are necessary to validate its effectiveness and define its place in clinical practice. The current research will offer important insights into the viability of citicoline as a standard therapeutic option for glaucoma patients.

### 3.6. Nicotinamide

Nicotinamide, also known as vitamin B3 or NAM, has a simple chemical structure consisting of a pyridine ring with an amide group at the 3-position. This structure allows it to serve as a precursor to nicotinamide adenine dinucleotide (NAD), a vital coenzyme in cellular metabolic processes. NAD is essential for mitochondrial function, as it supports oxidative phosphorylation and ATP production, which are critical for maintaining neuronal health and energy homeostasis. As cellular concentrations of NAD decline with age, mitochondrial dysfunction, inflammation, and oxidative stress worsen, leading to neurodegeneration [113,114,115]. Nicotinamide replenishes NAD levels, supporting mitochondrial function and cellular energy metabolism, while also influencing calcium homeostasis, vascular regulation, and neuronal health, making it a promising candidate for neuroprotection in glaucoma [116,117,118].

A study revealed that the reduced retinal NAD levels caused by aging in mice could be mitigated by high-dose oral nicotinamide [119,120]. Another study showed that oral nicotinamide inhibits RGC loss and nuclear shrinkage [121]. These studies showed that nicotinamide protected RGCs from glaucomatous damage by improving mitochondrial health and reducing oxidative stress. A recent study demonstrated that a combination of NADPH and NAC synergistically reduced apoptosis, axonal damage, and peroxidation in retinal ganglion cells while inhibiting gliosis and p38/MAPK activation in Müller cells, suggesting their potential as a neuroprotective and anti-inflammatory treatment for glaucoma [122]. Novel delivery methods were also investigated and demonstrated a positive result [123]. Similar findings were observed with genetic approaches to overexpress NAD-producing enzymes, such as nicotinamide mononucleotide adenylyl transferase 1 (NMNAT1), NMNAT2, and NMNAT3 [120,124,125]. On the other hand, epigallocatechin gallate (EGCG), a polyphenol found in green tea, promotes NAD production through a mechanism dependent on NMN and NMNAT2, making it a useful compound for developing new drugs [125].

Human studies have also supported the potential of nicotinamide in glaucoma treatment. A study reported significantly reduced plasma NAD levels in patients with primary POAG compared to controls, suggesting a systemic NAD deficiency in glaucoma [126]. This finding aligns with preclinical data and underscores the need for therapeutic strategies to restore NAD levels. A randomized crossover clinical trial (ACTRN12617000809336) showed that oral nicotinamide supplementation enhanced RGC function, as assessed by ERG, regardless of IOP levels [127]. Additionally, a Phase II randomized clinical trial (NCT03797469) found short-term improvements in visual function in patients with moderate OAG who received a combination of nicotinamide and pyruvate [128].

Ongoing clinical trials are exploring the long-term effects of nicotinamide supplementation in glaucoma patients. The Glaucoma Nicotinamide Trial (TGNT) is investigating the neuroprotective benefits of nicotinamide in 660 OAG patients, with an anticipated completion date by the end of 2026 (NCT05275738). Additionally, a Phase III RCT is evaluating the effect of oral nicotinamide on visual field progression over 27 months, with results expected by 2026 (NCT05405868). Another study is examining the neuroprotective potential of nicotinamide riboside, a more bioavailable precursor, over 24 months in 125 POAG patients [129,130]. As research progresses, nicotinamide could become an integral part of glaucoma management, offering hope for improved patient outcomes.

### 3.7. Insulin

The mTOR pathway is crucial for RGC energy metabolism, with insulin serving as a primary activator of both mTORC1 and mTORC2 [131]. Insulin crosses the blood-brain/retinal barrier and impacts neuronal survival, neurotransmission, and glucose uptake. Impaired insulin signaling is associated with neurodegenerative diseases, including glaucoma [132]. The research indicates that insulin enhances glucose transport, promotes dendritic regeneration, and supports neuronal survival. The activation of mTORC1/2 is vital for insulin-mediated dendrite repair and synapse restoration in RGCs, positioning insulin as a promising therapeutic target [133].

Intranasal insulin has emerged as a promising method for bypassing the blood–brain barrier without causing systemic side effects like hypoglycemia [134]. Research on intranasal insulin in Alzheimer’s and mild cognitive impairment shows that it is a safe and effective way to target insulin signaling in neurodegeneration [135]. Although clinical trials in glaucoma are yet to be conducted, preclinical evidence suggests that exogenous insulin may help preserve RGCs [132]. A Phase 1 trial investigating topical insulin eye drops for glaucoma was conducted and showed safety in patients with glaucoma [136]. Overall, intranasal and topical insulin presents a promising treatment strategy for neurodegenerative diseases, though further research is required to validate its efficacy.

### 3.8. Resveratrol

Resveratrol (RES), a polyphenolic compound with antioxidant properties, has shown potential in slowing glaucoma progression by supporting RGC health [137]. RES stimulates cell growth, reduces apoptosis, and alleviates oxidative stress, especially in RGCs exposed to hydrogen peroxide [138].

Additionally, RES protects RGC axons by inhibiting JNK protein phosphorylation through Sirt1 activation and prevents oxidative stress-related damage by suppressing the MAPK pathway [139]. RES preserves retinal function by modulating hypoxia-inducible factor-1 alpha (HIF-1α), vascular endothelial growth factor (VEGF), and the p38/p53 pathways, while concurrently activating the PI3K/Akt pathway to promote cell survival [140]. These mechanisms demonstrate RES’s therapeutic potential in protecting RGCs from degeneration and managing glaucoma progression [141]. Intravitreal administration of RES successfully protected RGCs from high IOP-induced cell death, though further research is needed to fully evaluate its efficacy [142].

### 3.9. Other Novel Drugs

Recent advancements in neuroprotective therapies for glaucoma have highlighted several promising compounds and mechanisms.

Dopamine (DA), produced by dopaminergic amacrine cells (DACs), plays key roles in light adaptation and circadian rhythms [143]. Increasing DA release from DACs, along with overexpressing the Drd1 receptor in all RGCs, significantly improves RGC survival, encourages axon regeneration after optic nerve damage, and protects vision in glaucoma models [144]. Future research using high-throughput single-cell sequencing will be important for understanding the cellular pathways activated by DRD1 [145].

Rho kinase (ROCK) inhibitors, such as ripasudil and fasudil, have shown promising neuroprotective effects in the treatment of glaucoma. These inhibitors not only reduce IOP by enhancing aqueous humor outflow through the trabecular meshwork but also exhibit neuroprotective properties by improving blood flow [146] to the optic nerve and promoting the survival of RGCs [147].

Omidenepag (OMD), an E prostanoid receptor 2 (EP2) agonist, demonstrated neuroprotective effects by suppressing excitotoxic RGC death and modulating glia–neuron interactions via cAMP signaling pathways [148]. The bioprecursor prodrug 10β,17β-dihydroxyestra-1,4-dien-3-one (DHED) selectively generates 17β-estradiol (E2) in the retina following topical application, effectively protecting RGCs and their axons in a male rat glaucoma model without raising systemic E2 levels [149]. The P2X7 receptor antagonist A740003 provided neuroprotection by inhibiting microglial activation, reducing retinal inflammation, and enhancing RGC survival in a chronic intraocular hypertension model [150].

Irisin, an exercise-induced myokine, showed neuroprotective properties by attenuating neuroinflammation and promoting autophagy through integrin αVβ5/AMPK signaling in an acute ocular hypertension model [151]. The novel small molecule H7E, an HDAC8 inhibitor, protected against glaucoma damage by inhibiting Müller glial activation and preventing retinal cell death from oxidative stress [152]. Finally, the Gramine derivative ITH12657 demonstrated significant neuroprotection against excitotoxicity-induced RGC death in a rat model, with specific protection observed in Brn3a+ RGCs and α-ONs-RGCs [153].

Collectively, these studies underscore the potential of diverse neuroprotective strategies in mitigating glaucomatous damage and preserving vision.

## 4. Emerging Technologies

### 4.1. Stem Cell Therapy

Stem cell therapy is a promising new approach for treating neurodegenerative diseases like glaucoma. It provides a dual therapeutic benefit by regenerating RGCs and differentiating them into new cell types, while also creating a neurotrophic environment that supports damaged RGCs [154,155].

Mesenchymal stem cells (MSCs), which are multipotent and can differentiate into various cell types such as neurons and glial cells, offer neuroprotective and regenerative effects. They promote neuronal growth, regulate inflammation and immune responses, stimulate angiogenesis, and help reduce demyelination and apoptosis [156,157,158]. MSCs also secrete platelet-derived growth factor (PDGF) and neurotrophic factors (NTFs) like CNTF, FGF-2, GDNF, neuritin, and BDNF, which enhance cell survival and foster the development of other cells [155,159,160,161,162].

Animal models and preclinical studies have shown that MSCs are effective in promoting RGC survival, reducing RGC loss, boosting growth factor expression, enhancing anti-inflammatory properties, and protecting trabecular meshwork tissue when administered intravitreally or intracamerally [160,163,164,165,166,167,168,169]. However, despite these encouraging results in animal models, clinical trials have encountered difficulties. For example, one trial found no significant improvement in visual performance after the intravitreal injection of autologous bone marrow-derived MSCs in a patient with advanced glaucoma. Additionally, another participant in the study developed retinal detachment with proliferative vitreoretinopathy, raising safety concerns [170]. Currently, the Intravitreal Mesenchymal Stem Cell Transplantation in Advanced Glaucoma Study (NCT02330978), a Phase I trial, is assessing the safety of intravitreal MSC injections in advanced glaucoma patients. Other trials, such as the Stem Cell Ophthalmology Treatment Study and its follow-up (NCT01920867, NCT03011541), are examining the variability in patient outcomes based on different MSC delivery techniques.

Human embryonic stem cells (hESCs) are pluripotent and can differentiate into any cell type, making them a potential source for RGCs [171]. Experimental studies have developed protocols for differentiating hESCs into RGCs, which have shown successful integration and the mediation of light responses in preclinical studies involving rat and monkey eyes [172,173,174]. However, the use of hESCs is ethically controversial and scientifically challenging. Other types of stem cells, such as oligodendrocyte precursor cells (OPCs), human neuronal progenitor cells, and retinal stem cells, have also demonstrated potential in protecting RGCs in animal models [175,176]. Mouse-induced pluripotent stem cell (miPSC)/mouse embryonic stem cell (mESC)-derived RGC and spermatogonial stem cell-derived RGC were also investigated [177,178]. Adipose tissue-derived regenerative cells, with their multipotent properties, are being evaluated in clinical trials (NCT02144103) for their safety and efficacy in glaucoma treatment [179].

Despite the potential neuroprotective abilities of stem cells, safety issues remain a significant concern. It is important to carefully evaluate the balance between graft survival and the risk of tumor development, as prolonged stem cell survival raises the likelihood of tumor formation. Moreover, the implanted cells might secrete harmful agents, affecting the microenvironment of RGCs [180]. Adverse effects, including reactive gliosis, vitreous clumping, extensive inflammation, and epiretinal membrane thickening are also concerns [181].

Despite the challenges, stem cell therapy holds significant potential for glaucoma treatment. Ongoing research aims to improve the safety and effectiveness of these treatments. Future investigations should prioritize ensuring the survival and function of transplanted cells, supporting their integration into retinal and brain networks, and maintaining long-term safety. Continued research and larger clinical trials are essential to fully realize the potential of stem cell therapy in glaucoma treatment, paving the way for its integration into clinical practice.

### 4.2. Gene Therapy

Gene therapy has become a promising strategy for the neuroprotection and treatment of glaucoma. Although currently restricted in clinical use, experimental studies and preclinical research have shown significant potential for gene therapy in managing and potentially reversing glaucomatous damage.

Gene therapy strategies for glaucoma focus on modifying specific genes associated with the disease’s pathogenesis and delivering neuroprotective factors to preserve RGCs. Studies have demonstrated that clustered regularly interspaced short-palindromic repeat (CRISPR)-mediated genome editing of the myocilin (MYOC) gene effectively lowers IOP and inhibits glaucomatous damage in mouse models [182]. Another genetic target under investigation is the tunica interna endothelial cell kinase (TEK), which plays a role in Schlemm’s canal development [183,184]. Additionally, genetic constructs designed to overexpress neuroprotective factors such as BDNF and its receptor TrkB have shown significant neuroprotective effects, enhancing RGC survival and preserving visual function in rodent models. This effect may prove to be a feasible therapeutic strategy in the planned Phase I/II trials [185,186,187,188,189]. In addition to BDNF, other neurotrophic factors such as CNTF have been encoded into genetic constructs to support RGC survival [45,190,191,192].

Other gene targets of different functions have also been investigated [193]. A study reactivating calcium/calmodulin-stimulated protein kinase II (CaMKII) activity in diseased mice resulted in the protection of RGCs and the preservation of visual function [156]. The overexpression of the complement C3 inhibitor shows a neuroprotective effect of RGC axons and somata [194]. The transduction of VEGF variants by VEGFR2 and PI3K signaling promotes synaptogenesis and increases the length of neurites and axons [195]. Combining AAV-γ-synuclein (mScng) promotor with CRISPR/Cas9 gene editing knocks down pro-degenerative genes, preserving the acutely injured RGC somata and axons [196]. The overexpression of Bcl-XL attenuates both RGC soma pathology and axonal degeneration in the optic nerve [197]. The overexpression of NMNAT1 as well as NMNAT2 restores the decreased NAD levels, showing a significant neuroprotective effect of RGC soma, axons, and the maintenance of visual function [120,198]. The overexpression of myc-associated protein X (MAX) prevents RGC death and protects optic nerve axons [199]. The overexpression of the X-linked inhibitor of apoptosis (XIAP) blocks the activation of apoptosis, providing both functional and structural protection of RGCs [200]. The overexpression of SOD2 reduced malonaldehyde, protecting RGCs from oxidative stress [201]. The overexpression of the adaptor molecule Protrudin significantly enhances central nervous system regeneration by promoting the accumulation of essential growth molecules and organelles in distal axons, both in vitro and in vivo. This regeneration is facilitated by Protrudin’s ability to link axonal organelles, motors, and membranes, highlighting its potential as a therapeutic target for axonal injury [202]. NFATc4 knockout in mice enhances RGC survival and delays axonal degeneration following optic nerve injury by suppressing pro-apoptotic signaling, highlighting NFATc4 as a potential therapeutic target for optic neuropathies [203]. Tau overexpression exacerbates retinal degeneration, while Tau silencing offers significant protection, highlighting the critical role of Tau in retinal integrity and its potential as a therapeutic target in glaucoma [204].

Despite promising results in preclinical models, gene therapy for glaucoma faces several challenges. The multifactorial and polygenic nature of glaucoma complicates the identification of effective genetic targets. Additionally, issues related to gene transfer efficiency, site-specific binding, and the potential for mutagenesis need to be addressed. Whole-genome sequencing and advancements in genome editing technology hold the potential to identify new therapeutic targets and refine existing gene therapy approaches. The development of more efficient viral vectors and delivery methods is crucial for translating preclinical successes into clinical practice. With continued advancements in genetic research and therapy, gene therapy may lead to breakthroughs in preserving vision and improving the quality of life for glaucoma patients.

### 4.3. Mitochondrial-Targeted Therapies and Transplantation

Mitochondria are essential for bioenergetics and play key roles in calcium regulation, cell signaling, apoptosis, and synaptic support. RGCs, with their large dendritic trees and unmyelinated axons in the retina, rely heavily on mitochondria to meet their high metabolic demands. As a result, these cells are highly susceptible to mitochondrial dysfunction, as seen in disorders like Leber hereditary optic neuropathy (LHON) and autosomal dominant optic atrophy [205] In glaucoma models, mitochondrial abnormalities appear before optic nerve degeneration, suggesting that metabolic stress plays a key role in RGC injury and degeneration [206].

Various neuroprotective, mitochondria-targeted therapies for glaucoma have been proposed, including dietary modifications, antioxidant supplementation, stem cell therapy, gene therapy, and mitochondrial transplantation [207]. Citicoline supports mitochondrial function, potentially preventing cell damage, promoting the growth of axons and dendrites, and improving dopaminergic and cholinergic activity [208]. Nicotinamide, through its role in regulating metabolism, has shown significant protection against optic nerve degeneration in animal models of glaucoma [209]. Though clinical evidence for metabolic therapies in glaucoma is limited due to the disease’s slow progression, small studies suggest neuroprotective benefits [128,210].

Mitochondrial transplantation, or mitotherapy, involves transferring functional mitochondria to cells with mitochondrial dysfunction, offering potential treatment for diseases linked to mitochondrial impairment [211]. Studies have shown promising results in animal models, including enhanced oxidative metabolism, neuroprotection, and axon regrowth in RGCs [212]. Human in vitro studies have also demonstrated mitotherapy’s ability to restore cellular function in diseases like LHON [213]. The success of mitochondrial transplantation is influenced by factors like the source and delivery method of the mitochondria, both of which require further investigation.

### 4.4. Nanotechnologies

Exosomes and nanoparticles are emerging as significant tools in neuroprotective and regenerative strategies for glaucoma treatment. Exosomes, extracellular vesicles released by different cell types, range from 30 to 150 nm in diameter and carry a variety of biologically active substances such as proteins, mRNA, miRNAs, and lipids. They act as intracellular signaling organelles and are involved in numerous pathological processes, including nerve injury and repair, vascular regeneration, and immune response. Their potential therapeutic applications in glaucoma are being increasingly recognized [214,215].

Exosomes derived from MSCs have demonstrated significant neuroprotective effects in glaucoma models. Studies using rat models revealed that bone marrow MSC (BMSC)-derived exosomes significantly increased the survival and axonal regeneration of RGCs through miRNA-dependent mechanisms [166,216]. These exosomes integrated into the inner retinal layers, highlighting the crucial role of miRNAs in their neuroprotective benefits. Additionally, human umbilical cord MSC-derived exosomes have been reported to promote RGC survival and glial activation in rat models [217]. These exosomes also preserved RGCs in human retinal explants post-axotomy by releasing growth factors, especially PDGF [160]. Another recent study demonstrates that small extracellular vesicles (sEVs) derived from MSCs overexpressing microRNA-22-3p (miR22) protect retinal RGCs from apoptosis and preserve retinal function in an NMDA-induced RGC injury model by inhibiting the MAPK signaling pathway. The findings suggest that miR22-sEVs could be a promising therapeutic approach for glaucoma and other diseases involving RGC damage [218]. Despite these promising findings, treatments involving BMSC-derived exosomes and human MSC-derived exosomes have been associated with extensive gliosis and inflammation, necessitating further research to optimize their therapeutic potential and minimize adverse effects [169].

Nanoparticles offer another promising approach for neuroprotection in glaucoma. These tiny particles can encapsulate drugs, siRNA, mRNA, or other nucleic acids, providing a hydrophobic environment that improves the solubility of drugs with low water solubility. Nanoparticles protect encapsulated drugs from hydrolytic degradation and improve their transport across biological barriers, making them effective drug carriers. The use of pharmaceutical nanoparticles also reduces the side effects associated with traditional topical treatments [219].

However, most nano eye drops designed for neuroprotection are still in the animal experimental stage. A study developed magnetic nanoparticles conjugated with neurotrophins. These nanoparticles protect neurotrophins from rapid degradation and facilitate their localization in the retina [220]. Other studies on rodent models suggested that GDNF delivered by nanoparticles could serve as a neuroprotective tool for treating glaucomatous optic neuropathy [221,222]. Another study demonstrates that circular RNA (circRNA)-based therapy using lipid nanoparticle (LNP)-formulated circNGF provides prolonged NGF protein expression and superior protection for RGCs compared to traditional NGF protein therapy, without retinal toxicity [223]. The other study created a topical formulation using tocopheryl polyethylene glycol succinate (TPGS) and Pluronic F127 curcumin nanocarriers, which demonstrated neuroprotective effects in rodent models of ocular hypertension, optic nerve disease, and partial optic nerve transection [224]. Additionally, a study explores the development of polymeric microparticles to sustain the release of hydrogen sulfide (H_2_S) for treating glaucoma. The research identifies critical process parameters that affect particle size, entrapment efficiency, and release profile, successfully optimizing a formulation that provides sustained H_2_S release for up to 30 days, with potential for both ocular hypotensive and neuroprotective effects [225]. Another study demonstrated that NAM-loaded extracellular vesicles (NAM-EVs) can effectively deliver NAM to RGCs, providing a neuroprotective effect and maintaining the health and function of RGCs [123]. The other study investigated the use of liposomal carriers to enhance the delivery and therapeutic efficacy of citicoline in treating glaucoma, showing promising results for improved bioavailability and sustained drug release in ocular applications [112].

Exosomes and nanoparticles represent innovative and promising strategies for neuroprotection and regeneration in glaucoma treatment. Further human studies are essential to fully elucidate the therapeutic potential of these technologies and optimize their applications in clinical settings, ultimately aiming to improve outcomes for glaucoma patients.

## 5. Conclusions

Glaucoma continues to pose a major global health challenge, requiring the development of innovative neuroprotective strategies to complement existing IOP-lowering treatments. Pharmacological interventions such as brimonidine, neurotrophic factors, memantine, Ginkgo biloba extract, citicoline, nicotinamide, insulin, and resveratrol have demonstrated promising neuroprotective effects in preclinical studies, with some showing positive results in early clinical trials. Emerging technologies, including stem cell therapy, gene therapy, mitochondrial-targeted therapies, and nanotechnologies, offer innovative approaches to neuroprotection and RGC regeneration. Despite the promising potential of these strategies, further research is required to address the challenges associated with their clinical application. Larger, long-term clinical trials are essential to confirm the efficacy and safety of these treatments, ensuring their successful integration into clinical practice.

The outlook for neuroprotective therapies in glaucoma is promising, but significant hurdles remain in translating preclinical success into routine clinical use. As these novel therapies continue to be explored, it is likely that targeted treatments combining pharmacological agents and cutting-edge technologies could become part of a comprehensive glaucoma management plan. In the future, personalized medicine approaches based on genetic and molecular profiling may further refine these treatments, offering more tailored and effective solutions for preventing vision loss in glaucoma patients.

## Abbreviations

RGCs	Retinal Ganglion Cells
IOP	Intraocular Pressure
BDNF	Brain-Derived Neurotrophic Factor
CNTF	Ciliary Neurotrophic Factor
NMDA	N-Methyl-D-Aspartate
ROS	Reactive Oxygen Species
TNF-α	Tumor Necrosis Factor-Alpha
FGF	Fibroblast Growth Factor
RNFL	Retinal Nerve Fiber Layer
NTG	Normal Tension Glaucoma
POAG	Primary Open-Angle Glaucoma
AH	Aqueous Humor
MAPK	Mitogen-Activated Protein Kinase
NGF	Nerve Growth Factor
p75NTR	p75 Neurotrophin Receptor
rhNGF	Recombinant Human Nerve Growth Factor
VEGF	Vascular Endothelial Growth Factor
NAD	Nicotinamide Adenine Dinucleotide
NADPH	Nicotinamide Adenine Dinucleotide Phosphate Hydrogen
DA	Dopamine
DACs	Dopaminergic Amacrine Cells
DRD1	Dopamine Receptor D1
ROCK	Rho-Associated Protein Kinase
EP2	E Prostanoid Receptor 2
CAMKII	Calcium/Calmodulin-Dependent Protein Kinase II
CRISPR	Clustered Regularly Interspaced Short Palindromic Repeats
MYOC	Myocilin
TEK	Tunica Interna Endothelial Cell Kinase
AAV	Adeno-Associated Virus
XIAP	X-Linked Inhibitor of Apoptosis
SOD2	Superoxide Dismutase 2
LHON	Leber Hereditary Optic Neuropathy
MSCs	Mesenchymal Stem Cells
PDGF	Platelet-Derived Growth Factor
hESCs	Human Embryonic Stem Cells
OPCs	Oligodendrocyte Precursor Cells
miPSC	Mouse-Induced Pluripotent Stem Cell
mESC	Mouse Embryonic Stem Cell
BMSC	Bone Marrow Mesenchymal Stem Cells
sEVs	Small Extracellular Vesicles
LNP	Lipid Nanoparticle
TPGS	Tocopheryl Polyethylene Glycol Succinate
EVs	Extracellular Vesicles

## References

[1] Tham Y.C., Li X., Wong T.Y., Quigley H.A., Aung T., Cheng C.Y. (2014). Global prevalence of glaucoma and projections of glaucoma burden through 2040: A systematic review and meta-analysis. Ophthalmology.

[2] Casson R.J. (2006). Possible role of excitotoxicity in the pathogenesis of glaucoma. Clin. Exp. Ophthalmol..

[3] Fan Gaskin J.C., Shah M.H., Chan E.C. (2021). Oxidative Stress and the Role of NADPH Oxidase in Glaucoma. Antioxidants.

[4] Vohra R., Tsai J.C., Kolko M. (2013). The role of inflammation in the pathogenesis of glaucoma. Surv. Ophthalmol..

[5] Chen S.D., Wang L., Zhang X.L. (2013). Neuroprotection in glaucoma: Present and future. Chin. Med. J..

[6] Pasutto F., Matsumoto T., Mardin C.Y., Sticht H., Brandstatter J.H., Michels-Rautenstrauss K., Weisschuh N., Gramer E., Ramdas W.D., van Koolwijk L.M. (2009). Heterozygous NTF4 mutations impairing neurotrophin-4 signaling in patients with primary open-angle glaucoma. Am. J. Hum. Genet..

[7] Malik J.M., Shevtsova Z., Bahr M., Kugler S. (2005). Long-term in vivo inhibition of CNS neurodegeneration by Bcl-XL gene transfer. Mol. Ther..

[8] Krupin T., Liebmann J.M., Greenfield D.S., Ritch R., Gardiner S., Low-Pressure Glaucoma Study Group (2011). A randomized trial of brimonidine versus timolol in preserving visual function: Results from the Low-Pressure Glaucoma Treatment Study. Am. J. Ophthalmol..

[9] De Moraes C.G., Liebmann J.M., Greenfield D.S., Gardiner S.K., Ritch R., Krupin T., Low-pressure Glaucoma Treatment Study Group (2012). Risk factors for visual field progression in the low-pressure glaucoma treatment study. Am. J. Ophthalmol..

[10] Goldberg J.L., Beykin G., Satterfield K.R., Nunez M., Lam B.L., Albini T.A. (2023). Phase I NT-501 Ciliary Neurotrophic Factor Implant Trial for Primary Open-Angle Glaucoma: Safety, Neuroprotection, and Neuroenhancement. Ophthalmol. Sci..

[11] Beykin G., Stell L., Halim M.S., Nunez M., Popova L., Nguyen B.T., Groth S.L., Dennis A., Li Z., Atkins M. (2022). Phase 1b Randomized Controlled Study of Short Course Topical Recombinant Human Nerve Growth Factor (rhNGF) for Neuroenhancement in Glaucoma: Safety, Tolerability, and Efficacy Measure Outcomes. Am. J. Ophthalmol..

[12] Weinreb R.N., Liebmann J.M., Cioffi G.A., Goldberg I., Brandt J.D., Johnson C.A., Zangwill L.M., Schneider S., Badger H., Bejanian M. (2018). Oral Memantine for the Treatment of Glaucoma: Design and Results of 2 Randomized, Placebo-Controlled, Phase 3 Studies. Ophthalmology.

[13] Quaranta L., Bettelli S., Uva M.G., Semeraro F., Turano R., Gandolfo E. (2003). Effect of Ginkgo biloba extract on preexisting visual field damage in normal tension glaucoma. Ophthalmology.

[14] Lee J., Sohn S.W., Kee C. (2013). Effect of Ginkgo biloba extract on visual field progression in normal tension glaucoma. J. Glaucoma.

[15] Guo X., Kong X., Huang R., Jin L., Ding X., He M., Liu X., Patel M.C., Congdon N.G. (2014). Effect of Ginkgo biloba on visual field and contrast sensitivity in Chinese patients with normal tension glaucoma: A randomized, crossover clinical trial. Investig. Ophthalmol. Vis. Sci..

[16] Guo X., Namekata K., Kimura A., Noro T., Azuchi Y., Semba K., Harada C., Yoshida H., Mitamura Y., Harada T. (2015). Brimonidine suppresses loss of retinal neurons and visual function in a murine model of optic neuritis. Neurosci. Lett..

[17] Lambert W.S., Ruiz L., Crish S.D., Wheeler L.A., Calkins D.J. (2011). Brimonidine prevents axonal and somatic degeneration of retinal ganglion cell neurons. Mol. Neurodegener..

[18] Lindsey J.D., Duong-Polk K.X., Hammond D., Chindasub P., Leung C.K., Weinreb R.N. (2015). Differential protection of injured retinal ganglion cell dendrites by brimonidine. Investig. Ophthalmol. Vis. Sci..

[19] Lafuente M.P., Villegas-Perez M.P., Mayor S., Aguilera M.E., Miralles de Imperial J., Vidal-Sanz M. (2002). Neuroprotective effects of brimonidine against transient ischemia-induced retinal ganglion cell death: A dose response in vivo study. Exp. Eye Res..

[20] Vidal-Sanz M., Lafuente M.P., Mayor-Torroglosa S., Aguilera M.E., Miralles de Imperial J., Villegas-Perez M.P. (2001). Brimonidine’s neuroprotective effects against transient ischaemia-induced retinal ganglion cell death. Eur. J. Ophthalmol..

[21] Chrysostomou V., Rezania F., Trounce I.A., Crowston J.G. (2013). Oxidative stress and mitochondrial dysfunction in glaucoma. Curr. Opin. Pharmacol..

[22] Goldenberg-Cohen N., Dadon-Bar-El S., Hasanreisoglu M., Avraham-Lubin B.C., Dratviman-Storobinsky O., Cohen Y., Weinberger D. (2009). Possible neuroprotective effect of brimonidine in a mouse model of ischaemic optic neuropathy. Clin. Exp. Ophthalmol..

[23] Aktas Z., Gurelik G., Akyurek N., Onol M., Hasanreisoglu B. (2007). Neuroprotective effect of topically applied brimonidine tartrate 0.2% in endothelin-1-induced optic nerve ischaemia model. Clin. Exp. Ophthalmol..

[24] Gao H., Qiao X., Cantor L.B., WuDunn D. (2002). Up-regulation of brain-derived neurotrophic factor expression by brimonidine in rat retinal ganglion cells. Arch. Ophthalmol..

[25] Jung K.I., Kim J.H., Han J.S., Park C.K. (2024). Exploring Neuroprotective Effects of Topical Brimonidine in Experimental Diabetic Retinopathy. Vivo.

[26] Semba K., Namekata K., Kimura A., Harada C., Mitamura Y., Harada T. (2014). Brimonidine prevents neurodegeneration in a mouse model of normal tension glaucoma. Cell Death Dis..

[27] Jung K.I., Kim J.H., Park C.K. (2015). alpha2-Adrenergic modulation of the glutamate receptor and transporter function in a chronic ocular hypertension model. Eur. J. Pharmacol..

[28] Lee D., Kim K.Y., Noh Y.H., Chai S., Lindsey J.D., Ellisman M.H., Weinreb R.N., Ju W.K. (2012). Brimonidine blocks glutamate excitotoxicity-induced oxidative stress and preserves mitochondrial transcription factor a in ischemic retinal injury. PLoS ONE.

[29] Lee K.Y., Nakayama M., Aihara M., Chen Y.N., Araie M. (2010). Brimonidine is neuroprotective against glutamate-induced neurotoxicity, oxidative stress, and hypoxia in purified rat retinal ganglion cells. Mol. Vis..

[30] Goldblum D., Kipfer-Kauer A., Sarra G.M., Wolf S., Frueh B.E. (2007). Distribution of amyloid precursor protein and amyloid-beta immunoreactivity in DBA/2J glaucomatous mouse retinas. Investig. Ophthalmol. Vis. Sci..

[31] Nizari S., Guo L., Davis B.M., Normando E.M., Galvao J., Turner L.A., Bizrah M., Dehabadi M., Tian K., Cordeiro M.F. (2016). Non-amyloidogenic effects of alpha2 adrenergic agonists: Implications for brimonidine-mediated neuroprotection. Cell. Death Dis..

[32] Feke G.T., Bex P.J., Taylor C.P., Rhee D.J., Turalba A.V., Chen T.C., Wand M., Pasquale L.R. (2014). Effect of brimonidine on retinal vascular autoregulation and short-term visual function in normal tension glaucoma. Am. J. Ophthalmol..

[33] Lonngren U., Napankangas U., Lafuente M., Mayor S., Lindqvist N., Vidal-Sanz M., Hallbook F. (2006). The growth factor response in ischemic rat retina and superior colliculus after brimonidine pre-treatment. Brain Res. Bull..

[34] Ben Simon G.J., Bakalash S., Aloni E., Rosner M. (2006). A rat model for acute rise in intraocular pressure: Immune modulation as a therapeutic strategy. Am. J. Ophthalmol..

[35] Namekata K., Noro T., Nishijima E., Sotozono A., Guo X., Harada C., Shinozaki Y., Mitamura Y., Nakano T., Harada T. (2024). Drug combination of topical ripasudil and brimonidine enhances neuroprotection in a mouse model of optic nerve injury. J. Pharmacol. Sci..

[36] Otsubo M., Sase K., Tsukahara C., Fujita N., Arizono I., Tokuda N., Kitaoka Y. (2024). Axonal protection by combination of ripasudil and brimonidine with upregulation of p-AMPK in TNF-induced optic nerve degeneration. Int. Ophthalmol..

[37] Evans D.W., Hosking S.L., Gherghel D., Bartlett J.D. (2003). Contrast sensitivity improves after brimonidine therapy in primary open angle glaucoma: A case for neuroprotection. Br. J. Ophthalmol..

[38] Tsai J.C., Chang H.W. (2005). Comparison of the effects of brimonidine 0.2% and timolol 0.5% on retinal nerve fiber layer thickness in ocular hypertensive patients: A prospective, unmasked study. J. Ocul. Pharmacol. Ther..

[39] Scuteri D., Bagetta G., Nucci C., Aiello F., Cesareo M., Tonin P., Corasaniti M.T. (2020). Evidence on the neuroprotective properties of brimonidine in glaucoma. Prog. Brain Res..

[40] Kimura A., Namekata K., Guo X., Harada C., Harada T. (2016). Neuroprotection, Growth Factors and BDNF-TrkB Signalling in Retinal Degeneration. Int. J. Mol. Sci..

[41] Chang E.E., Goldberg J.L. (2012). Glaucoma 2.0: Neuroprotection, neuroregeneration, neuroenhancement. Ophthalmology.

[42] Saragovi H.U., Hamel E., Di Polo A. (2009). A neurotrophic rationale for the therapy of neurodegenerative disorders. Curr. Alzheimer Res..

[43] Lambiase A., Aloe L., Centofanti M., Parisi V., Bao S.N., Mantelli F., Colafrancesco V., Manni G.L., Bucci M.G., Bonini S. (2009). Experimental and clinical evidence of neuroprotection by nerve growth factor eye drops: Implications for glaucoma. Proc. Natl. Acad. Sci. USA.

[44] Oddone F., Roberti G., Micera A., Busanello A., Bonini S., Quaranta L., Agnifili L., Manni G. (2017). Exploring Serum Levels of Brain Derived Neurotrophic Factor and Nerve Growth Factor Across Glaucoma Stages. PLoS ONE.

[45] Pease M.E., Zack D.J., Berlinicke C., Bloom K., Cone F., Wang Y., Klein R.L., Hauswirth W.W., Quigley H.A. (2009). Effect of CNTF on retinal ganglion cell survival in experimental glaucoma. Investig. Ophthalmol. Vis. Sci..

[46] Grozdanic S.D., Lazic T., Kuehn M.H., Harper M.M., Kardon R.H., Kwon Y.H., Lavik E.B., Sakaguchi D.S. (2010). Exogenous modulation of intrinsic optic nerve neuroprotective activity. Graefes Arch. Clin. Exp. Ophthalmol..

[47] Parrilla-Reverter G., Agudo M., Sobrado-Calvo P., Salinas-Navarro M., Villegas-Perez M.P., Vidal-Sanz M. (2009). Effects of different neurotrophic factors on the survival of retinal ganglion cells after a complete intraorbital nerve crush injury: A quantitative in vivo study. Exp. Eye Res..

[48] Weber A.J., Viswanathan S., Ramanathan C., Harman C.D. (2010). Combined application of BDNF to the eye and brain enhances ganglion cell survival and function in the cat after optic nerve injury. Investig. Ophthalmol. Vis. Sci..

[49] Flachsbarth K., Jankowiak W., Kruszewski K., Helbing S., Bartsch S., Bartsch U. (2018). Pronounced synergistic neuroprotective effect of GDNF and CNTF on axotomized retinal ganglion cells in the adult mouse. Exp. Eye Res..

[50] Hameed S.S., Bodi N.E., Miller R.C., Sharma T.P. (2024). Neuritin 1 Drives Therapeutic Preservation of Retinal Ganglion Cells in an Ex Vivo Human Glaucoma Model. J. Ocul. Pharmacol. Ther..

[51] Johnson T.V., Bull N.D., Martin K.R. (2011). Neurotrophic factor delivery as a protective treatment for glaucoma. Exp. Eye Res..

[52] Vecino E., Garcia-Crespo D., Garcia M., Martinez-Millan L., Sharma S.C., Carrascal E. (2002). Rat retinal ganglion cells co-express brain derived neurotrophic factor (BDNF) and its receptor TrkB. Vis. Res..

[53] Cha Y.W., Kim S.T. (2021). Serum and aqueous humor levels of brain-derived neurotrophic factor in patients with primary open-angle glaucoma and normal-tension glaucoma. Int. Ophthalmol..

[54] Uzel M.M., Elgin U., Boral B., Cicek M., Sen E., Sener B., Yilmazbas P. (2018). The effect of trabeculectomy on serum brain-derived neurotrophic factor levels in primary open-angle glaucoma. Graefes Arch. Clin. Exp. Ophthalmol..

[55] Lazaldin M.A.M., Iezhitsa I., Agarwal R., Agarwal P., Ismail N.M. (2023). Neuroprotective effects of exogenous brain-derived neurotrophic factor on amyloid-beta 1-40-induced retinal degeneration. Neural Regen. Res..

[56] Binley K.E., Ng W.S., Barde Y.A., Song B., Morgan J.E. (2016). Brain-derived neurotrophic factor prevents dendritic retraction of adult mouse retinal ganglion cells. Eur. J. Neurosci..

[57] Shpak A.A., Guekht A.B., Druzhkova T.A., Kozlova K.I., Gulyaeva N.V. (2017). Ciliary neurotrophic factor in patients with primary open-angle glaucoma and age-related cataract. Mol. Vis..

[58] Boulton T.G., Stahl N., Yancopoulos G.D. (1994). Ciliary neurotrophic factor/leukemia inhibitory factor/interleukin 6/oncostatin M family of cytokines induces tyrosine phosphorylation of a common set of proteins overlapping those induced by other cytokines and growth factors. J. Biol. Chem..

[59] Escartin C., Pierre K., Colin A., Brouillet E., Delzescaux T., Guillermier M., Dhenain M., Deglon N., Hantraye P., Pellerin L. (2007). Activation of astrocytes by CNTF induces metabolic plasticity and increases resistance to metabolic insults. J. Neurosci..

[60] Lee K., Choi J.O., Hwang A., Bae H.W., Kim C.Y. (2022). Ciliary Neurotrophic Factor Derived From Astrocytes Protects Retinal Ganglion Cells Through PI3K/AKT, JAK/STAT, and MAPK/ERK Pathways. Investig. Ophthalmol. Vis. Sci..

[61] Leibinger M., Andreadaki A., Diekmann H., Fischer D. (2013). Neuronal STAT3 activation is essential for CNTF- and inflammatory stimulation-induced CNS axon regeneration. Cell Death Dis..

[62] Muller A., Hauk T.G., Leibinger M., Marienfeld R., Fischer D. (2009). Exogenous CNTF stimulates axon regeneration of retinal ganglion cells partially via endogenous CNTF. Mol. Cell Neurosci..

[63] Mey J., Thanos S. (1993). Intravitreal injections of neurotrophic factors support the survival of axotomized retinal ganglion cells in adult rats in vivo. Brain Res..

[64] Cui Q., Yip H.K., Zhao R.C., So K.F., Harvey A.R. (2003). Intraocular elevation of cyclic AMP potentiates ciliary neurotrophic factor-induced regeneration of adult rat retinal ganglion cell axons. Mol. Cell Neurosci..

[65] Bertrand J., Winton M.J., Rodriguez-Hernandez N., Campenot R.B., McKerracher L. (2005). Application of Rho antagonist to neuronal cell bodies promotes neurite growth in compartmented cultures and regeneration of retinal ganglion cell axons in the optic nerve of adult rats. J. Neurosci..

[66] Lingor P., Tonges L., Pieper N., Bermel C., Barski E., Planchamp V., Bahr M. (2008). ROCK inhibition and CNTF interact on intrinsic signalling pathways and differentially regulate survival and regeneration in retinal ganglion cells. Brain.

[67] Kauper K., McGovern C., Sherman S., Heatherton P., Rapoza R., Stabila P., Dean B., Lee A., Borges S., Bouchard B. (2012). Two-year intraocular delivery of ciliary neurotrophic factor by encapsulated cell technology implants in patients with chronic retinal degenerative diseases. Investig. Ophthalmol. Vis. Sci..

[68] Talcott K.E., Ratnam K., Sundquist S.M., Lucero A.S., Lujan B.J., Tao W., Porco T.C., Roorda A., Duncan J.L. (2011). Longitudinal study of cone photoreceptors during retinal degeneration and in response to ciliary neurotrophic factor treatment. Investig. Ophthalmol. Vis. Sci..

[69] Zhang K., Hopkins J.J., Heier J.S., Birch D.G., Halperin L.S., Albini T.A., Brown D.M., Jaffe G.J., Tao W., Williams G.A. (2011). Ciliary neurotrophic factor delivered by encapsulated cell intraocular implants for treatment of geographic atrophy in age-related macular degeneration. Proc. Natl. Acad. Sci. USA.

[70] Tirassa P., Rosso P., Iannitelli A. (2018). Ocular Nerve Growth Factor (NGF) and NGF Eye Drop Application as Paradigms to Investigate NGF Neuroprotective and Reparative Actions. Methods Mol. Biol..

[71] Chen Q., Wang H., Liao S., Gao Y., Liao R., Little P.J., Xu J., Feng Z.P., Zheng Y., Zheng W. (2015). Nerve growth factor protects retinal ganglion cells against injury induced by retinal ischemia-reperfusion in rats. Growth. Factors.

[72] Patapoutian A., Reichardt L.F. (2001). Trk receptors: Mediators of neurotrophin action. Curr. Opin. Neurobiol..

[73] Colafrancesco V., Parisi V., Sposato V., Rossi S., Russo M.A., Coassin M., Lambiase A., Aloe L. (2011). Ocular application of nerve growth factor protects degenerating retinal ganglion cells in a rat model of glaucoma. J. Glaucoma.

[74] Guo L., Davis B.M., Ravindran N., Galvao J., Kapoor N., Haamedi N., Shamsher E., Luong V., Fico E., Cordeiro M.F. (2020). Topical recombinant human Nerve growth factor (rh-NGF) is neuroprotective to retinal ganglion cells by targeting secondary degeneration. Sci. Rep..

[75] Lebrun-Julien F., Bertrand M.J., De Backer O., Stellwagen D., Morales C.R., Di Polo A., Barker P.A. (2010). ProNGF induces TNFalpha-dependent death of retinal ganglion cells through a p75NTR non-cell-autonomous signaling pathway. Proc. Natl. Acad. Sci. USA.

[76] Mesentier-Louro L.A., Rosso P., Carito V., Mendez-Otero R., Santiago M.F., Rama P., Lambiase A., Tirassa P. (2019). Nerve Growth Factor Role on Retinal Ganglion Cell Survival and Axon Regrowth: Effects of Ocular Administration in Experimental Model of Optic Nerve Injury. Mol. Neurobiol..

[77] Ferrari M.P., Mantelli F., Sacchetti M., Antonangeli M.I., Cattani F., D’Anniballe G., Sinigaglia F., Ruffini P.A., Lambiase A. (2014). Safety and pharmacokinetics of escalating doses of human recombinant nerve growth factor eye drops in a double-masked, randomized clinical trial. BioDrugs.

[78] Parsons C.G., Stoffler A., Danysz W. (2007). Memantine: A NMDA receptor antagonist that improves memory by restoration of homeostasis in the glutamatergic system–too little activation is bad, too much is even worse. Neuropharmacology.

[79] Osborne N.N. (2009). Recent clinical findings with memantine should not mean that the idea of neuroprotection in glaucoma is abandoned. Acta Ophthalmol..

[80] Hare W.A., WoldeMussie E., Lai R.K., Ton H., Ruiz G., Chun T., Wheeler L. (2004). Efficacy and safety of memantine treatment for reduction of changes associated with experimental glaucoma in monkey, I: Functional measures. Investig. Ophthalmol. Vis. Sci..

[81] Seki M., Lipton S.A. (2008). Targeting excitotoxic/free radical signaling pathways for therapeutic intervention in glaucoma. Prog. Brain Res..

[82] Yucel Y.H., Gupta N., Zhang Q., Mizisin A.P., Kalichman M.W., Weinreb R.N. (2006). Memantine protects neurons from shrinkage in the lateral geniculate nucleus in experimental glaucoma. Arch. Ophthalmol..

[83] Kim T.W., Kim D.M., Park K.H., Kim H. (2002). Neuroprotective effect of memantine in a rabbit model of optic nerve ischemia. Korean J. Ophthalmol..

[84] Burgoyne C.F. (2015). The non-human primate experimental glaucoma model. Exp. Eye Res..

[85] Wamsley S., Gabelt B.T., Dahl D.B., Case G.L., Sherwood R.W., May C.A., Hernandez M.R., Kaufman P.L. (2005). Vitreous glutamate concentration and axon loss in monkeys with experimental glaucoma. Arch. Ophthalmol..

[86] Levkovitch-Verbin H., Quigley H.A., Kerrigan-Baumrind L.A., D’Anna S.A., Kerrigan D., Pease M.E. (2001). Optic nerve transection in monkeys may result in secondary degeneration of retinal ganglion cells. Investig. Ophthalmol. Vis. Sci..

[87] Vianna J.R., Chauhan B.C. (2015). How to detect progression in glaucoma. Prog. Brain Res..

[88] Eckert A., Keil U., Scherping I., Hauptmann S., Muller W.E. (2005). Stabilization of mitochondrial membrane potential and improvement of neuronal energy metabolism by Ginkgo biloba extract EGb 761. Ann. N. Y. Acad. Sci..

[89] Ritch R. (2000). Potential role for Ginkgo biloba extract in the treatment of glaucoma. Med. Hypotheses.

[90] Kang J.M., Lin S. (2018). Ginkgo biloba and its potential role in glaucoma. Curr. Opin. Ophthalmol..

[91] Hirooka K., Tokuda M., Miyamoto O., Itano T., Baba T., Shiraga F. (2004). The Ginkgo biloba extract (EGb 761) provides a neuroprotective effect on retinal ganglion cells in a rat model of chronic glaucoma. Curr. Eye Res..

[92] Ma K., Xu L., Zhan H., Zhang S., Pu M., Jonas J.B. (2009). Dosage dependence of the effect of Ginkgo biloba on the rat retinal ganglion cell survival after optic nerve crush. Eye.

[93] Ma K., Xu L., Zhang H., Zhang S., Pu M., Jonas J.B. (2010). The effect of ginkgo biloba on the rat retinal ganglion cell survival in the optic nerve crush model. Acta Ophthalmol..

[94] Harris A., Gross J., Moore N., Do T., Huang A., Gama W., Siesky B. (2018). The effects of antioxidants on ocular blood flow in patients with glaucoma. Acta Ophthalmol..

[95] Park J.W., Kwon H.J., Chung W.S., Kim C.Y., Seong G.J. (2011). Short-term effects of Ginkgo biloba extract on peripapillary retinal blood flow in normal tension glaucoma. Korean J. Ophthalmol..

[96] Faiq M.A., Wollstein G., Schuman J.S., Chan K.C. (2019). Cholinergic nervous system and glaucoma: From basic science to clinical applications. Prog. Retin. Eye Res..

[97] Roberti G., Tanga L., Michelessi M., Quaranta L., Parisi V., Manni G., Oddone F. (2015). Cytidine 5’-Diphosphocholine (Citicoline) in Glaucoma: Rationale of Its Use, Current Evidence and Future Perspectives. Int. J. Mol. Sci..

[98] Adibhatla R.M., Hatcher J.F., Dempsey R.J. (2001). Effects of citicoline on phospholipid and glutathione levels in transient cerebral ischemia. Stroke.

[99] van der Merwe Y., Yang X., Ho L.C., Yu Y., Chau Y., Leung C.K.-S., Conner I.P., Steketee M.B., Wollstein G., Schuman J.S. (2016). Citicoline preserves optic nerve integrity and visuomotor function following chronic intraocular pressure elevation. Investig. Ophthalmol. Vis. Sci..

[100] Virno M., Pecori-Giraldi J., Liguori A., De Gregorio F. (2000). The protective effect of citicoline on the progression of the perimetric defects in glaucomatous patients (perimetric study with a 10-year follow-up). Acta Ophthalmol. Scand. Suppl..

[101] Parisi V. (2005). Electrophysiological assessment of glaucomatous visual dysfunction during treatment with cytidine-5’-diphosphocholine (citicoline): A study of 8 years of follow-up. Doc. Ophthalmol..

[102] Rejdak R., Toczolowski J., Kurkowski J., Kaminski M.L., Rejdak K., Stelmasiak Z., Grieb P. (2003). Oral citicoline treatment improves visual pathway function in glaucoma. Med. Sci. Monit..

[103] Parisi V., Coppola G., Centofanti M., Oddone F., Angrisani A.M., Ziccardi L., Ricci B., Quaranta L., Manni G. (2008). Evidence of the neuroprotective role of citicoline in glaucoma patients. Prog. Brain Res..

[104] Ottobelli L., Manni G.L., Centofanti M., Iester M., Allevena F., Rossetti L. (2013). Citicoline oral solution in glaucoma: Is there a role in slowing disease progression?. Ophthalmologica.

[105] Rossetti L., Iester M., Tranchina L., Ottobelli L., Coco G., Calcatelli E., Ancona C., Cirafici P., Manni G. (2020). Can Treatment With Citicoline Eyedrops Reduce Progression in Glaucoma? The Results of a Randomized Placebo-controlled Clinical Trial. J. Glaucoma.

[106] Parisi V., Centofanti M., Ziccardi L., Tanga L., Michelessi M., Roberti G., Manni G. (2015). Treatment with citicoline eye drops enhances retinal function and neural conduction along the visual pathways in open angle glaucoma. Graefes Arch. Clin. Exp. Ophthalmol..

[107] Parisi V., Oddone F., Roberti G., Tanga L., Carnevale C., Ziccardi L., Manni G. (2019). Enhancement of Retinal Function and of Neural Conduction Along the Visual Pathway Induced by Treatment with Citicoline Eye Drops in Liposomal Formulation in Open Angle Glaucoma: A Pilot Electrofunctional Study. Adv. Ther..

[108] Roberti G., Tanga L., Parisi V., Sampalmieri M., Centofanti M., Manni G. (2014). A preliminary study of the neuroprotective role of citicoline eye drops in glaucomatous optic neuropathy. Indian J. Ophthalmol..

[109] Grieb P., Junemann A., Rekas M., Rejdak R. (2016). Citicoline: A Food Beneficial for Patients Suffering from or Threated with Glaucoma. Front. Aging Neurosci..

[110] Anton A., Garcia V., Munoz M., Gonzales K., Ayala E., Del Mar Sanchez E., Morilla-Grasa A. (2022). The Effect of Oral Citicoline and Docosahexaenoic Acid on the Visual Field of Patients with Glaucoma: A Randomized Trial. Life.

[111] Marino P.F., Rossi G.C.M., Campagna G., Capobianco D., Costagliola C., on behalf of QUALICOS Study Group (2020). Effects of Citicoline, Homotaurine, and Vitamin E on Contrast Sensitivity and Visual-Related Quality of Life in Patients with Primary Open-Angle Glaucoma: A Preliminary Study. Molecules.

[112] Bonechi C., Mahdizadeh F.F., Talarico L., Pepi S., Tamasi G., Leone G., Consumi M., Donati A., Magnani A. (2023). Liposomal Encapsulation of Citicoline for Ocular Drug Delivery. Int. J. Mol. Sci..

[113] Verdin E. (2015). NAD(+) in aging, metabolism, and neurodegeneration. Science.

[114] Cimaglia G., Votruba M., Morgan J.E., Andre H., Williams P.A. (2020). Potential Therapeutic Benefit of NAD(+) Supplementation for Glaucoma and Age-Related Macular Degeneration. Nutrients.

[115] Zhang M., Ying W. (2019). NAD(+) Deficiency Is a Common Central Pathological Factor of a Number of Diseases and Aging: Mechanisms and Therapeutic Implications. Antioxid Redox Signal.

[116] Araie M., Mayama C. (2011). Use of calcium channel blockers for glaucoma. Prog. Retin. Eye Res..

[117] Pasquale L.R. (2016). Vascular and autonomic dysregulation in primary open-angle glaucoma. Curr. Opin. Ophthalmol..

[118] Resch H., Garhofer G., Fuchsjager-Mayrl G., Hommer A., Schmetterer L. (2009). Endothelial dysfunction in glaucoma. Acta Ophthalmol..

[119] Williams P.A., Harder J.M., Foxworth N.E., Cardozo B.H., Cochran K.E., John S.W.M. (2017). Nicotinamide and WLD(S) Act Together to Prevent Neurodegeneration in Glaucoma. Front. Neurosci..

[120] Williams P.A., Harder J.M., Foxworth N.E., Cochran K.E., Philip V.M., Porciatti V., Smithies O., John S.W. (2017). Vitamin B(3) modulates mitochondrial vulnerability and prevents glaucoma in aged mice. Science.

[121] Tribble J.R., Otmani A., Sun S., Ellis S.A., Cimaglia G., Vohra R., Joe M., Lardner E., Venkataraman A.P., Dominguez-Vicent A. (2021). Nicotinamide provides neuroprotection in glaucoma by protecting against mitochondrial and metabolic dysfunction. Redox Biol.

[122] Yu N., Wu X., Zhang C., Qin Q., Gu Y., Ke W., Liu X., Zhang Q., Liu Z., Chen M. (2024). NADPH and NAC synergistically inhibits chronic ocular hypertension-induced neurodegeneration and neuroinflammation through regulating p38/MAPK pathway and peroxidation. Biomed. Pharmacother..

[123] Kim M., Kim J.Y., Rhim W.K., Cimaglia G., Want A., Morgan J.E., Williams P.A., Park C.G., Han D.K., Rho S. (2024). Extracellular vesicle encapsulated nicotinamide delivered via a trans-scleral route provides retinal ganglion cell neuroprotection. Acta Neuropathol. Commun..

[124] Felici R., Lapucci A., Ramazzotti M., Chiarugi A. (2013). Insight into molecular and functional properties of NMNAT3 reveals new hints of NAD homeostasis within human mitochondria. PLoS ONE.

[125] Tribble J.R., Joe M., Varricchio C., Otmani A., Canovai A., Habchi B., Daskalakis E., Chaleckis R., Loreto A., Gilley J. (2024). NMNAT2 is a druggable target to drive neuronal NAD production. Nat. Commun..

[126] Kouassi Nzoughet J., Chao de la Barca J.M., Guehlouz K., Leruez S., Coulbault L., Allouche S., Bocca C., Muller J., Amati-Bonneau P., Gohier P. (2019). Nicotinamide Deficiency in Primary Open-Angle Glaucoma. Investig. Ophthalmol. Vis. Sci..

[127] Hui F., Tang J., Williams P.A., McGuinness M.B., Hadoux X., Casson R.J., Coote M., Trounce I.A., Martin K.R., van Wijngaarden P. (2020). Improvement in inner retinal function in glaucoma with nicotinamide (vitamin B3) supplementation: A crossover randomized clinical trial. Clin. Exp. Ophthalmol..

[128] De Moraes C.G., John S.W.M., Williams P.A., Blumberg D.M., Cioffi G.A., Liebmann J.M. (2022). Nicotinamide and Pyruvate for Neuroenhancement in Open-Angle Glaucoma: A Phase 2 Randomized Clinical Trial. JAMA Ophthalmol..

[129] Leung C.K.S., Ren S.T., Chan P.P.M., Wan K.H.N., Kam A.K.W., Lai G.W.K., Chiu V.S.M., Ko M.W.L., Yiu C.K.F., Yu M.C.Y. (2022). Nicotinamide riboside as a neuroprotective therapy for glaucoma: Study protocol for a randomized, double-blind, placebo-control trial. Trials.

[130] Mehmel M., Jovanovic N., Spitz U. (2020). Nicotinamide Riboside-The Current State of Research and Therapeutic Uses. Nutrients.

[131] Di Polo A. (2015). Dendrite pathology and neurodegeneration: Focus on mTOR. Neural Regen. Res..

[132] Agostinone J., Alarcon-Martinez L., Gamlin C., Yu W.Q., Wong R.O.L., Di Polo A. (2018). Insulin signalling promotes dendrite and synapse regeneration and restores circuit function after axonal injury. Brain.

[133] Jhanwar-Uniyal M., Wainwright J.V., Mohan A.L., Tobias M.E., Murali R., Gandhi C.D., Schmidt M.H. (2019). Diverse signaling mechanisms of mTOR complexes: mTORC1 and mTORC2 in forming a formidable relationship. Adv. Biol. Regul..

[134] Avgerinos K.I., Kalaitzidis G., Malli A., Kalaitzoglou D., Myserlis P.G., Lioutas V.A. (2018). Intranasal insulin in Alzheimer’s dementia or mild cognitive impairment: A systematic review. J. Neurol..

[135] Craft S., Baker L.D., Montine T.J., Minoshima S., Watson G.S., Claxton A., Arbuckle M., Callaghan M., Tsai E., Plymate S.R. (2012). Intranasal insulin therapy for Alzheimer disease and amnestic mild cognitive impairment: A pilot clinical trial. Arch. Neurol..

[136] Wennberg Smith Z., Beykin G., Saludares M., Nunez M., Wang Q., Di Polo A., Goldberg J.L. (2024). A Phase 1 Trial of Topical Insulin for Patients with Glaucoma. Investig. Ophthalmol. Vis. Sci..

[137] Pezzuto J.M. (2019). Resveratrol: Twenty Years of Growth, Development and Controversy. Biomol. Ther..

[138] Pang Y., Qin M., Hu P., Ji K., Xiao R., Sun N., Pan X., Zhang X. (2020). Resveratrol protects retinal ganglion cells against ischemia induced damage by increasing Opa1 expression. Int. J. Mol. Med..

[139] Wu Y., Pang Y., Wei W., Shao A., Deng C., Li X., Chang H., Hu P., Liu X., Zhang X. (2020). Resveratrol protects retinal ganglion cell axons through regulation of the SIRT1-JNK pathway. Exp. Eye Res..

[140] Ji K., Li Z., Lei Y., Xu W., Ouyang L., He T., Xing Y. (2021). Resveratrol attenuates retinal ganglion cell loss in a mouse model of retinal ischemia reperfusion injury via multiple pathways. Exp. Eye Res..

[141] Luo H., Zhuang J., Hu P., Ye W., Chen S., Pang Y., Li N., Deng C., Zhang X. (2018). Resveratrol Delays Retinal Ganglion Cell Loss and Attenuates Gliosis-Related Inflammation From Ischemia-Reperfusion Injury. Investig. Ophthalmol. Vis. Sci..

[142] Cao K., Ishida T., Fang Y., Shinohara K., Li X., Nagaoka N., Ohno-Matsui K., Yoshida T. (2020). Protection of the Retinal Ganglion Cells: Intravitreal Injection of Resveratrol in Mouse Model of Ocular Hypertension. Investig. Ophthalmol. Vis. Sci..

[143] Chen S., Zhi Z., Ruan Q., Liu Q., Li F., Wan F., Reinach P.S., Chen J., Qu J., Zhou X. (2017). Bright Light Suppresses Form-Deprivation Myopia Development With Activation of Dopamine D1 Receptor Signaling in the ON Pathway in Retina. Investig. Ophthalmol. Vis. Sci..

[144] Lin J., Xue J., Xu Q., Liu Z., Zhao C., Tang J., Han J., A S., Wang W., Zhuo Y. (2022). In situ-crosslinked hydrogel-induced experimental glaucoma model with persistent ocular hypertension and neurodegeneration. Biomater. Sci..

[145] Zhang Q., Xue J., Tang J., Wu S., Liu Z., Wu C., Liu C., Liu Y., Lin J., Han J. (2024). Modulating amacrine cell-derived dopamine signaling promotes optic nerve regeneration and preserves visual function. Sci. Adv..

[146] Ohta Y., Takaseki S., Yoshitomi T. (2017). Effects of ripasudil hydrochloride hydrate (K-115), a Rho-kinase inhibitor, on ocular blood flow and ciliary artery smooth muscle contraction in rabbits. Jpn. J. Ophthalmol..

[147] Tanna A.P., Johnson M. (2018). Rho Kinase Inhibitors as a Novel Treatment for Glaucoma and Ocular Hypertension. Ophthalmology.

[148] Nakamura N., Honjo M., Yamagishi-Kimura R., Sakata R., Watanabe S., Aihara M. (2024). Neuroprotective effect of omidenepag on excitotoxic retinal ganglion cell death regulating COX-2-EP2-cAMP-PKA/Epac pathway via Neuron-Glia interaction. Neuroscience.

[149] Kapic A., Zaman K., Nguyen V., Neagu G.C., Sumien N., Prokai L., Prokai-Tatrai K. (2024). The Prodrug DHED Delivers 17beta-Estradiol into the Retina for Protection of Retinal Ganglion Cells and Preservation of Visual Function in an Animal Model of Glaucoma. Cells.

[150] Zhu Y., Li S.Y., Zhang L.J., Lei B., Wang Y.C., Wang Z. (2024). Neuroprotection of the P2X7 receptor antagonist A740003 on retinal ganglion cells in experimental glaucoma. Neuroreport.

[151] Zhang Q., Xiang S., Chen X., Rong Y., Huang L., Chen Z., Yao K., Chen W., Deng C., Wang J. (2024). Irisin attenuates acute glaucoma-induced neuroinflammation by activating microglia-integrin alphaVbeta5/AMPK and promoting autophagy. Int. Immunopharmacol..

[152] Wu L.H., Cheng Y.W., Lin F.L., Hsu K.C., Wang M.H., Yen J.L., Wang T.J., Lin T.E., Liu Y.C., Huang W.J. (2024). A novel HDAC8 inhibitor H7E exerts retinoprotective effects against glaucomatous injury via ameliorating aberrant Muller glia activation and oxidative stress. Biomed. Pharmacother..

[153] Di Pierdomenico J., Gallego-Ortega A., Norte-Munoz M., Vidal-Villegas B., Bravo I., Boluda-Ruiz M., Bernal-Garro J.M., Fernandez-Bueno I., Pastor-Jimeno J.C., Villegas-Perez M.P. (2024). Evaluation of the neuroprotective efficacy of the gramine derivative ITH12657 against NMDA-induced excitotoxicity in the rat retina. Front. Neuroanat..

[154] Dahlmann-Noor A., Vijay S., Jayaram H., Limb A., Khaw P.T. (2010). Current approaches and future prospects for stem cell rescue and regeneration of the retina and optic nerve. Can. J. Ophthalmol..

[155] Fu L., Kwok S.S., Chan Y.K., Ming Lai J.S., Pan W., Nie L., Shih K.C. (2019). Therapeutic Strategies for Attenuation of Retinal Ganglion Cell Injury in Optic Neuropathies: Concepts in Translational Research and Therapeutic Implications. Biomed. Res. Int..

[156] Guo X., Zhou J., Starr C., Mohns E.J., Li Y., Chen E.P., Yoon Y., Kellner C.P., Tanaka K., Wang H. (2021). Preservation of vision after CaMKII-mediated protection of retinal ganglion cells. Cell.

[157] Bull N.D., Johnson T.V., Martin K.R. (2008). Stem cells for neuroprotection in glaucoma. Prog. Brain Res..

[158] Hu B.Y., Xin M., Chen M., Yu P., Zeng L.Z. (2024). Mesenchymal stem cells for repairing glaucomatous optic nerve. Int. J. Ophthalmol..

[159] Harrell C.R., Fellabaum C., Arsenijevic A., Markovic B.S., Djonov V., Volarevic V. (2019). Therapeutic Potential of Mesenchymal Stem Cells and Their Secretome in the Treatment of Glaucoma. Stem Cells Int..

[160] Johnson T.V., DeKorver N.W., Levasseur V.A., Osborne A., Tassoni A., Lorber B., Heller J.P., Villasmil R., Bull N.D., Martin K.R. (2014). Identification of retinal ganglion cell neuroprotection conferred by platelet-derived growth factor through analysis of the mesenchymal stem cell secretome. Brain.

[161] Park S.S., Moisseiev E., Bauer G., Anderson J.D., Grant M.B., Zam A., Zawadzki R.J., Werner J.S., Nolta J.A. (2017). Advances in bone marrow stem cell therapy for retinal dysfunction. Prog. Retin. Eye Res..

[162] Crigler L., Robey R.C., Asawachaicharn A., Gaupp D., Phinney D.G. (2006). Human mesenchymal stem cell subpopulations express a variety of neuro-regulatory molecules and promote neuronal cell survival and neuritogenesis. Exp. Neurol..

[163] Emre E., Yuksel N., Duruksu G., Pirhan D., Subasi C., Erman G., Karaoz E. (2015). Neuroprotective effects of intravitreally transplanted adipose tissue and bone marrow-derived mesenchymal stem cells in an experimental ocular hypertension model. Cytotherapy.

[164] Harper M.M., Grozdanic S.D., Blits B., Kuehn M.H., Zamzow D., Buss J.E., Kardon R.H., Sakaguchi D.S. (2011). Transplantation of BDNF-secreting mesenchymal stem cells provides neuroprotection in chronically hypertensive rat eyes. Investig. Ophthalmol. Vis. Sci..

[165] Johnson T.V., Bull N.D., Hunt D.P., Marina N., Tomarev S.I., Martin K.R. (2010). Neuroprotective effects of intravitreal mesenchymal stem cell transplantation in experimental glaucoma. Investig. Ophthalmol. Vis. Sci..

[166] Mead B., Amaral J., Tomarev S. (2018). Mesenchymal Stem Cell-Derived Small Extracellular Vesicles Promote Neuroprotection in Rodent Models of Glaucoma. Investig. Ophthalmol. Vis. Sci..

[167] Roubeix C., Godefroy D., Mias C., Sapienza A., Riancho L., Degardin J., Fradot V., Ivkovic I., Picaud S., Sennlaub F. (2015). Intraocular pressure reduction and neuroprotection conferred by bone marrow-derived mesenchymal stem cells in an animal model of glaucoma. Stem Cell Res. Ther..

[168] Wang Y., Lv J., Huang C., Li X., Chen Y., Wu W., Wu R. (2021). Human Umbilical Cord-Mesenchymal Stem Cells Survive and Migrate within the Vitreous Cavity and Ameliorate Retinal Damage in a Novel Rat Model of Chronic Glaucoma. Stem Cells Int..

[169] Osborne A., Sanderson J., Martin K.R. (2018). Neuroprotective Effects of Human Mesenchymal Stem Cells and Platelet-Derived Growth Factor on Human Retinal Ganglion Cells. Stem Cells.

[170] Vilela C.A.P., Messias A., Calado R.T., Siqueira R.C., Silva M.J.L., Covas D.T., Paula J.S. (2021). Retinal function after intravitreal injection of autologous bone marrow-derived mesenchymal stromal cells in advanced glaucoma. Doc. Ophthalmol..

[171] Lopez Sanchez M.I., Crowston J.G., Mackey D.A., Trounce I.A. (2016). Emerging Mitochondrial Therapeutic Targets in Optic Neuropathies. Pharmacol. Ther..

[172] Chao J.R., Lamba D.A., Klesert T.R., Torre A., Hoshino A., Taylor R.J., Jayabalu A., Engel A.L., Khuu T.H., Wang R.K. (2017). Transplantation of Human Embryonic Stem Cell-Derived Retinal Cells into the Subretinal Space of a Non-Human Primate. Transl. Vis. Sci. Technol..

[173] Sluch V.M., Davis C.H., Ranganathan V., Kerr J.M., Krick K., Martin R., Berlinicke C.A., Marsh-Armstrong N., Diamond J.S., Mao H.Q. (2015). Differentiation of human ESCs to retinal ganglion cells using a CRISPR engineered reporter cell line. Sci. Rep..

[174] Venugopalan P., Wang Y., Nguyen T., Huang A., Muller K.J., Goldberg J.L. (2016). Transplanted neurons integrate into adult retinas and respond to light. Nat. Commun..

[175] Ma J., Guo C., Guo C., Sun Y., Liao T., Beattie U., Lopez F.J., Chen D.F., Lashkari K. (2015). Transplantation of Human Neural Progenitor Cells Expressing IGF-1 Enhances Retinal Ganglion Cell Survival. PLoS ONE.

[176] Zhou X., Xia X.B., Xiong S.Q. (2013). Neuro-protection of retinal stem cells transplantation combined with copolymer-1 immunization in a rat model of glaucoma. Mol. Cell Neurosci..

[177] Oswald J., Kegeles E., Minelli T., Volchkov P., Baranov P. (2021). Transplantation of miPSC/mESC-derived retinal ganglion cells into healthy and glaucomatous retinas. Mol. Ther. Methods Clin. Dev..

[178] Suen H.C., Qian Y., Liao J., Luk C.S., Lee W.T., Ng J.K.W., Chan T.T.H., Hou H.W., Li I., Li K. (2019). Transplantation of Retinal Ganglion Cells Derived from Male Germline Stem Cell as a Potential Treatment to Glaucoma. Stem Cells Dev..

[179] Qin Y., Ge G., Yang P., Wang L., Qiao Y., Pan G., Yang H., Bai J., Cui W., Geng D. (2023). An Update on Adipose-Derived Stem Cells for Regenerative Medicine: Where Challenge Meets Opportunity. Adv. Sci..

[180] Greco S.J., Rameshwar P. (2008). Microenvironmental considerations in the application of human mesenchymal stem cells in regenerative therapies. Biologics.

[181] Kasetty M.A., Hedges T.R., Witkin A.J. (2022). Bilateral Epiretinal Membrane Formation after Intravitreal Injections of Autologous Mesenchymal Stem Cells. Retin. Cases Brief Rep..

[182] Jain A., Zode G., Kasetti R.B., Ran F.A., Yan W., Sharma T.P., Bugge K., Searby C.C., Fingert J.H., Zhang F. (2017). CRISPR-Cas9-based treatment of myocilin-associated glaucoma. Proc. Natl. Acad. Sci. USA.

[183] Souma T., Tompson S.W., Thomson B.R., Siggs O.M., Kizhatil K., Yamaguchi S., Feng L., Limviphuvadh V., Whisenhunt K.N., Maurer-Stroh S. (2016). Angiopoietin receptor TEK mutations underlie primary congenital glaucoma with variable expressivity. J. Clin. Investig..

[184] Thomson B.R., Heinen S., Jeansson M., Ghosh A.K., Fatima A., Sung H.K., Onay T., Chen H., Yamaguchi S., Economides A.N. (2014). A lymphatic defect causes ocular hypertension and glaucoma in mice. J. Clin. Investig..

[185] Osborne A., Khatib T.Z., Songra L., Barber A.C., Hall K., Kong G.Y.X., Widdowson P.S., Martin K.R. (2018). Neuroprotection of retinal ganglion cells by a novel gene therapy construct that achieves sustained enhancement of brain-derived neurotrophic factor/tropomyosin-related kinase receptor-B signaling. Cell Death Dis..

[186] Osborne A., Wang A.X.Z., Tassoni A., Widdowson P.S., Martin K.R. (2018). Design of a Novel Gene Therapy Construct to Achieve Sustained Brain-Derived Neurotrophic Factor Signaling in Neurons. Hum. Gene Ther..

[187] Khatib T.Z., Osborne A., Yang S., Ali Z., Jia W., Manyakin I., Hall K., Watt R., Widdowson P.S., Martin K.R. (2021). Receptor-ligand supplementation via a self-cleaving 2A peptide-based gene therapy promotes CNS axonal transport with functional recovery. Sci. Adv..

[188] Wojcik-Gryciuk A., Gajewska-Wozniak O., Kordecka K., Boguszewski P.M., Waleszczyk W., Skup M. (2020). Neuroprotection of Retinal Ganglion Cells with AAV2-BDNF Pretreatment Restoring Normal TrkB Receptor Protein Levels in Glaucoma. Int. J. Mol. Sci..

[189] Alqawlaq S., Sivak J.M., Huzil J.T., Ivanova M.V., Flanagan J.G., Beazely M.A., Foldvari M. (2014). Preclinical development and ocular biodistribution of gemini-DNA nanoparticles after intravitreal and topical administration: Towards non-invasive glaucoma gene therapy. Nanomedicine.

[190] Cen L.P., Liang J.J., Chen J.H., Harvey A.R., Ng T.K., Zhang M., Pang C.P., Cui Q., Fan Y.M. (2017). AAV-mediated transfer of RhoA shRNA and CNTF promotes retinal ganglion cell survival and axon regeneration. Neuroscience.

[191] Hellstrom M., Pollett M.A., Harvey A.R. (2011). Post-injury delivery of rAAV2-CNTF combined with short-term pharmacotherapy is neuroprotective and promotes extensive axonal regeneration after optic nerve trauma. J. Neurotrauma.

[192] Do Rhee K., Wang Y., Ten Hoeve J., Stiles L., Nguyen T.T.T., Zhang X., Vergnes L., Reue K., Shirihai O., Bok D. (2022). Ciliary neurotrophic factor-mediated neuroprotection involves enhanced glycolysis and anabolism in degenerating mouse retinas. Nat. Commun..

[193] Hakim A., Guido B., Narsineni L., Chen D.W., Foldvari M. (2023). Gene therapy strategies for glaucoma from IOP reduction to retinal neuroprotection: Progress towards non-viral systems. Adv. Drug. Deliv. Rev..

[194] Bosco A., Anderson S.R., Breen K.T., Romero C.O., Steele M.R., Chiodo V.A., Boye S.L., Hauswirth W.W., Tomlinson S., Vetter M.L. (2018). Complement C3-Targeted Gene Therapy Restricts Onset and Progression of Neurodegeneration in Chronic Mouse Glaucoma. Mol. Ther..

[195] Shen J., Xiao R., Bair J., Wang F., Vandenberghe L.H., Dartt D., Baranov P., Ng Y.S.E. (2018). Novel engineered, membrane-localized variants of vascular endothelial growth factor (VEGF) protect retinal ganglion cells: A proof-of-concept study. Cell Death Dis..

[196] Wang Q., Zhuang P., Huang H., Li L., Liu L., Webber H.C., Dalal R., Siew L., Fligor C.M., Chang K.C. (2020). Mouse gamma-Synuclein Promoter-Mediated Gene Expression and Editing in Mammalian Retinal Ganglion Cells. J. Neurosci..

[197] Donahue R.J., Fehrman R.L., Gustafson J.R., Nickells R.W. (2021). BCLX(L) gene therapy moderates neuropathology in the DBA/2J mouse model of inherited glaucoma. Cell Death Dis..

[198] Fang F., Zhuang P., Feng X., Liu P., Liu D., Huang H., Li L., Chen W., Liu L., Sun Y. (2022). NMNAT2 is downregulated in glaucomatous RGCs, and RGC-specific gene therapy rescues neurodegeneration and visual function. Mol. Ther..

[199] Lani-Louzada R., Marra C., Dias M.S., de Araujo V.G., Abreu C.A., Ribas V.T., Adesse D., Allodi S., Chiodo V., Hauswirth W. (2022). Neuroprotective Gene Therapy by Overexpression of the Transcription Factor MAX in Rat Models of Glaucomatous Neurodegeneration. Investig. Ophthalmol. Vis. Sci..

[200] Visuvanathan S., Baker A.N., Lagali P.S., Coupland S.G., Miller G., Hauswirth W.W., Tsilfidis C. (2022). XIAP gene therapy effects on retinal ganglion cell structure and function in a mouse model of glaucoma. Gene Ther..

[201] Jiang W., Tang L., Zeng J., Chen B. (2016). Adeno-associated virus mediated SOD gene therapy protects the retinal ganglion cells from chronic intraocular pressure elevation induced injury via attenuating oxidative stress and improving mitochondrial dysfunction in a rat model. Am. J. Transl. Res..

[202] Petrova V., Pearson C.S., Ching J., Tribble J.R., Solano A.G., Yang Y., Love F.M., Watt R.J., Osborne A., Reid E. (2020). Protrudin functions from the endoplasmic reticulum to support axon regeneration in the adult CNS. Nat. Commun..

[203] Mackiewicz J., Tomczak J., Lisek M., Sakowicz A., Guo F., Boczek T. (2024). NFATc4 Knockout Promotes Neuroprotection and Retinal Ganglion Cell Regeneration After Optic Nerve Injury. Mol. Neurobiol..

[204] Thananthirige K.P.M., Chitranshi N., Basavarajappa D., Rajput R., Abbasi M., Palanivel V., Gupta V.B., Paulo J.A., Koronyo-Hamaoui M., Mirzaei M. (2024). Tau modulation through AAV9 therapy augments Akt/Erk survival signalling in glaucoma mitigating the retinal degenerative phenotype. Acta Neuropathol. Commun..

[205] Brown M.D., Starikovskaya E., Derbeneva O., Hosseini S., Allen J.C., Mikhailovskaya I.E., Sukernik R.I., Wallace D.C. (2002). The role of mtDNA background in disease expression: A new primary LHON mutation associated with Western Eurasian haplogroup. J. Hum. Genet..

[206] Alexander C., Votruba M., Pesch U.E., Thiselton D.L., Mayer S., Moore A., Rodriguez M., Kellner U., Leo-Kottler B., Auburger G. (2000). OPA1, encoding a dynamin-related GTPase, is mutated in autosomal dominant optic atrophy linked to chromosome 3q28. Nat. Genet..

[207] Osborne N.N., Nunez-Alvarez C., Joglar B., Del Olmo-Aguado S. (2016). Glaucoma: Focus on mitochondria in relation to pathogenesis and neuroprotection. Eur. J. Pharmacol..

[208] Harun-Or-Rashid M., Pappenhagen N., Palmer P.G., Smith M.A., Gevorgyan V., Wilson G.N., Crish S.D., Inman D.M. (2018). Structural and Functional Rescue of Chronic Metabolically Stressed Optic Nerves through Respiration. J. Neurosci..

[209] Oddone F., Rossetti L., Parravano M., Sbardella D., Coletta M., Ziccardi L., Roberti G., Carnevale C., Romano D., Manni G. (2021). Citicoline in Ophthalmological Neurodegenerative Disease: A Comprehensive Review. Pharmaceuticals.

[210] Williams P.A., Harder J.M., Cardozo B.H., Foxworth N.E., John S.W.M. (2018). Nicotinamide treatment robustly protects from inherited mouse glaucoma. Commun. Integr. Biol..

[211] Lanza M., Gironi Carnevale U.A., Mele L., Bifani Sconocchia M., Bartollino S., Costagliola C. (2019). Morphological and Functional Evaluation of Oral Citicoline Therapy in Chronic Open-Angle Glaucoma Patients: A Pilot Study With a 2-Year Follow-Up. Front. Pharmacol..

[212] Gabelein C.G., Feng Q., Sarajlic E., Zambelli T., Guillaume-Gentil O., Kornmann B., Vorholt J.A. (2022). Mitochondria transplantation between living cells. PLoS Biol..

[213] Nascimento-Dos-Santos G., de-Souza-Ferreira E., Lani R., Faria C.C., Araujo V.G., Teixeira-Pinheiro L.C., Vasconcelos T., Goncalo T., Santiago M.F., Linden R. (2020). Neuroprotection from optic nerve injury and modulation of oxidative metabolism by transplantation of active mitochondria to the retina. Biochim. Biophys. Acta Mol. Basis Dis..

[214] Li S.F., Han Y., Wang F., Su Y. (2020). Progress in exosomes and their potential use in ocular diseases. Int. J. Ophthalmol..

[215] Lener T., Gimona M., Aigner L., Borger V., Buzas E., Camussi G., Chaput N., Chatterjee D., Court F.A., Del Portillo H.A. (2015). Applying extracellular vesicles based therapeutics in clinical trials—An ISEV position paper. J. Extracell Vesicles.

[216] Mead B., Tomarev S. (2017). Bone Marrow-Derived Mesenchymal Stem Cells-Derived Exosomes Promote Survival of Retinal Ganglion Cells Through miRNA-Dependent Mechanisms. Stem Cells Transl. Med..

[217] Pan D., Chang X., Xu M., Zhang M., Zhang S., Wang Y., Luo X., Xu J., Yang X., Sun X. (2019). UMSC-derived exosomes promote retinal ganglion cells survival in a rat model of optic nerve crush. J. Chem. Neuroanat..

[218] Yu B., Wang K., Hao H., Liu Y., Yue Y., Li X., Xing X., Zhang X. (2024). Small extracellular vesicles derived from microRNA-22-3p-overexpressing mesenchymal stem cells protect retinal ganglion cells by regulating MAPK pathway. Commun. Biol..

[219] Wang J.J., Zeng Z.W., Xiao R.Z., Xie T., Zhou G.L., Zhan X.R., Wang S.L. (2011). Recent advances of chitosan nanoparticles as drug carriers. Int. J. Nanomed..

[220] Giannaccini M., Usai A., Chiellini F., Guadagni V., Andreazzoli M., Ori M., Pasqualetti M., Dente L., Raffa V. (2018). Neurotrophin-conjugated nanoparticles prevent retina damage induced by oxidative stress. Cell Mol Life Sci..

[221] Jiang C., Moore M.J., Zhang X., Klassen H., Langer R., Young M. (2007). Intravitreal injections of GDNF-loaded biodegradable microspheres are neuroprotective in a rat model of glaucoma. Mol Vis.

[222] Checa-Casalengua P., Jiang C., Bravo-Osuna I., Tucker B.A., Molina-Martinez I.T., Young M.J., Herrero-Vanrell R. (2011). Retinal ganglion cells survival in a glaucoma model by GDNF/Vit E PLGA microspheres prepared according to a novel microencapsulation procedure. J. Control Release.

[223] Jiang W., Xiao D., Wu C., Yang J., Peng X., Chen L., Zhang J., Zha G., Li W., Ju R. (2024). Circular RNA-based therapy provides sustained and robust neuroprotection for retinal ganglion cells. Mol. Ther. Nucleic Acids.

[224] Davis B.M., Pahlitzsch M., Guo L., Balendra S., Shah P., Ravindran N., Malaguarnera G., Sisa C., Shamsher E., Hamze H. (2018). Topical Curcumin Nanocarriers are Neuroprotective in Eye Disease. Sci. Rep..

[225] Rai A., Mhatre S., Chandler C., Opere C., Singh S. (2024). Application of Quality by Design in the Development of Hydrogen Sulfide Donor Loaded Polymeric Microparticles. AAPS PharmSciTech.

